# Identifying transcriptomic profiles of iron–quercetin complex treated peripheral blood mononuclear cells from healthy volunteers and diabetic patients

**DOI:** 10.1038/s41598-024-60197-1

**Published:** 2024-04-24

**Authors:** Phattarawadee Innuan, Chonticha Sirikul, Nampeung Anukul, Gwenaël Rolin, Nathupakorn Dechsupa, Jiraporn Kantapan

**Affiliations:** 1 Molecular Imaging and Therapy Research Unit, Department of Radiologic Technology, Faculty of Associated Medical Sciences, Chiang Mai University, Chiang Mai, 50200 Thailand; 2 Department of Radiologic Technology, Faculty of Associated Medical Sciences, Chiang Mai University, Chiang Mai, 50200 Thailand; 3 Division of Transfusion Science, Department of Medical Technology, Faculty of Associated Medical Sciences, Chiang Mai University, Chiang Mai, 50200 Thailand; 4 INSERM CIC-1431, CHU Besançon, 25000 Besançon, France

**Keywords:** Iron–quercetin complex, Peripheral blood mononuclear cells (PBMCs), Cell therapy, Tissue repair, Regenerative medicine, Angiogenesis, Biotechnology, Biomaterials, Nanobiotechnology, Regenerative medicine

## Abstract

Peripheral blood is an alternative source of stem/progenitor cells for regenerative medicine owing to its ease of retrieval and blood bank storage. Previous in vitro studies indicated that the conditioned medium derived from peripheral blood mononuclear cells (PBMCs) treated with the iron–quercetin complex (IronQ) contains potent angiogenesis and wound-healing properties. This study aims to unveil the intricate regulatory mechanisms governing the effects of IronQ on the transcriptome profiles of human PBMCs from healthy volunteers and those with diabetes mellitus (DM) using RNA sequencing analysis. Our findings revealed 3741 and 2204 differentially expressed genes (DEGs) when treating healthy and DM PBMCs with IronQ, respectively. Functional enrichment analyses underscored the biological processes shared by the DEGs in both conditions, including inflammatory responses, cell migration, cellular stress responses, and angiogenesis. A comprehensive exploration of these molecular alterations exposed a network of 20 hub genes essential in response to stimuli, cell migration, immune processes, and the mitogen-activated protein kinase (MAPK) pathway. The activation of these pathways enabled PBMCs to potentiate angiogenesis and tissue repair. Corroborating this, quantitative real-time polymerase chain reaction (qRT-PCR) and cell phenotyping confirmed the upregulation of candidate genes associated with anti-inflammatory, pro-angiogenesis, and tissue repair processes in IronQ-treated PBMCs. In summary, combining IronQ and PBMCs brings about substantial shifts in gene expression profiles and activates pathways that are crucial for tissue repair and immune response, which is promising for the enhancement of the therapeutic potential of PBMCs, especially in diabetic wound healing.

## Introduction

Tissue repair is a fundamental biological process for maintaining homeostasis and proper function in multicellular organisms. However, in severe injuries and chronic diseases, the body's innate ability to regenerate and repair tissues may be limited, hindering the complete restoration of damaged structures^1^. Cell therapy is a pioneering strategy for overcoming these limitations; this approach involves introducing exogenous cells or stimulating the body's endogenous cells to facilitate more efficient and effective tissue repair^2^. Cell therapy targets specific cellular mechanisms and pathways to sustain, replace, or repair damaged tissues through cell transplantation, manipulation, or modification. Due to their remarkable regenerative capabilities, stem and progenitor cells are at the forefront of discussions in regenerative medicine^3^. Mesenchymal stem cells (MSCs) are particularly emphasized for their regenerative potential, and beyond their differentiation into various cell types, these transplanted cells can locally release a diverse array of pro-regenerative substances at physiologically relevant quantities^4^. However, conventional methods for isolating these stem/progenitor cells can be invasive. An encouraging alternative lies in peripheral blood, an easily accessible source of stem/progenitor cells. Peripheral blood is recognized for its harboring of stem/progenitor cells with regenerative potential comparable to that of MSCs, and these cells can be isolated through minimally invasive or non-invasive procedures, thus representing a more straightforward and patient-friendly approach to promoting the healing process^5,6^.

Peripheral blood mononuclear cells (PBMCs) encompass a diverse population of immune cells, including lymphocytes (T cells, B cells, and NK cells) and monocyte-derived progenitor cells, which are promising for restoring damaged tissues and promoting regeneration. Their significance lies in their versatile functionality, which ranges from immune responses to regenerative properties^7,8^. PBMCs exhibit considerable promise in tissue repair mechanisms, as they actively engage in angiogenesis, tissue remodeling, and wound healing processes. Blocki et al. shed light on the potential of spindle-shaped blood-derived angiogenic cells (BDACs) from peripheral blood cell cultures subjected to macromolecular crowding (MMC). These cells exhibited a remarkable ability to secrete a diverse array of pro-angiogenic factors, underscoring their robust angiogenic activity. In a murine hind-limb ischemia model, an injection of BDACs effectively rescued affected tissues by expediting and enhancing revascularization^9^. Furthermore, when a nucleated cell fraction obtained from peripheral blood was cocultured with stem cell feeder cells, this gave rise to mesenchymal-like cells, which were termed “blood-derived mesenchymal stem cells” (BD-MSCs). In a mouse model, BD-MSCs were used to treat critical-sized calvarial bone defects, which markedly improved bone healing outcomes^10^. Paracrine factors have demonstrated significant efficacy in wound healing mechanisms, marking a paradigm shift from sole reliance on stem cell transplantation in regenerative therapies^11^. Researchers have increasingly recognized the potential of utilizing stem cells' diverse bioactive molecules. This complex mixture, which is collectively known as the secretome, holds immense therapeutic promise by modulating cellular processes, spurring tissue regeneration, and exerting immunomodulatory effects^12^. Notably, the secretome derived from apoptotic PBMCs has shown heightened production of cytokines and chemokines—notably, IL8 and VEGF^13^. These findings have highlighted the accelerated wound healing potential of the PBMC secretome in mouse models of full-thickness skin wounds in vivo, along with demonstrating enhanced migration of human primary keratinocytes and fibroblasts and increased proliferation of endothelial cells in vitro^14^. Beyond cutaneous wound healing, the secretome derived from apoptotic PBMCs has revealed therapeutic potential in animal models of myocardial infarction^15^. Furthermore, in experimental animal models of autoimmune myocarditis, the PBMC secretome exhibited promising anti-inflammatory effects, effectively attenuating myocardial inflammation^16^.

We previously developed a methodology for cultivating myeloid angiogenic cells derived from human PBMCs by using a paramagnetic agent called the iron–quercetin complex, which is referred to as IronQ. Following a 10-day culture period, the resultant cells exhibited a spindle-shaped morphology and expressed critical markers associated with angiogenesis, including CD11b, CD31, VEGFR2, and CD105. Additionally, they demonstrated the secretion of various cytokines and growth factors in a similar fashion to other blood-derived angiogenic cells. Interestingly, these cells displayed a pronounced pro-angiogenic potential and actively promoted fibroblast migration in vitro^17^. The paramagnetic IronQ has shown significant promise as a versatile theranostic agent, as it is both a contrast agent for magnetic resonance imaging and an invigorating therapeutic agent. This agent has demonstrated safety in cell tracking and therapy^18^. Given that our IronQ-PBMC-derived angiogenic cells originated from a readily accessible source and exhibited renewable tissue properties, as well as their cultivation using a versatile theranostic agent that was capable of stimulating PBMCs and enabling cell tracking via MRI, these cells hold tremendous promise for use in tissue repair strategies.

Diabetes, a chronic metabolic disorder, is often associated with delayed wound healing^19^. Autologous cell-based therapy represents a promising frontier in the pursuit of adequate wound healing, particularly in individuals grappling with the complications of diabetes. This therapeutic approach involves harnessing a patient's cells, which makes it attractive due to its potential for enhanced biocompatibility and reduced risk of immune rejection. This promising process involves utilizing autologous CD34^+^ cells obtained from peripheral blood, which is stimulated by granulocyte-colony-stimulating factor (G‐CSF). This method has shown safety and efficacy in addressing chronic non-healing ulcers in diabetic patients^20^. However, clinical trials involving freshly isolated CD34^+^ cells have faced significant cell isolation and expansion challenges^21^. Moreover, diabetic patients experience a marked reduction in both the number and functional capacity of progenitor cells, significantly hampering the clinical potential of autologous cell therapy^22,23^. Addressing these functional deficits and augmenting cell counts to a sufficient level is pivotal for the success of autologous cell therapy in diabetic patients. As mentioned earlier regarding the stimulating properties of IronQ in enhancing the therapeutic efficacy of healthy PBMCs, in this study, we also hypothesized that IronQ could rejuvenate the functionalities of human PBMCs obtained from patients with diabetes. We anticipated that these resulting cells (PBMCs‐IronQ) would demonstrate tissue reparative capacity.

This innovative contrast agent (IronQ) has been extensively studied and has shown promising results in various biomedical applications, including cell tracking and active biological interventions for promoting cell and tissue regeneration^17,18,24^. However, the interactions that affect transcriptome profiles and the underlying molecular mechanisms that enhance the therapeutic properties of PBMCs still need to be fully understood. In this study, we explored the interactions between IronQ and PBMCs and delved into transcriptome analysis to uncover alterations in gene expression and understand the activated cellular pathways. Our investigation revealed extensive modifications in the gene expression profile following IronQ treatment, which is a novel finding that has not been previously documented. Moreover, the analysis of gene expression patterns in IronQ-treated PBMCs unveiled significant vital genes and gene ontology (GO) pathways associated with anti-inflammatory and tissue repair processes—specifically, in angiogenesis and wound healing. The RNA-seq results were substantiated through quantitative real-time polymerase chain reaction (qRT-PCR) and cell phenotyping with immunocytometric techniques, providing compelling evidence of IronQ's capacity to modulate biological pathways in PBMCs and influence their therapeutic characteristics. Our findings delve into the immense potential of PBMCs for therapeutic applications, the exploration of their immunomodulatory properties, their role in tissue repair, and their applications in cell-based therapies to address a wide range of advanced regenerative medicine.

## Results

### Baseline characteristics of the sample and RNA sequencing information

**Figure 1:**
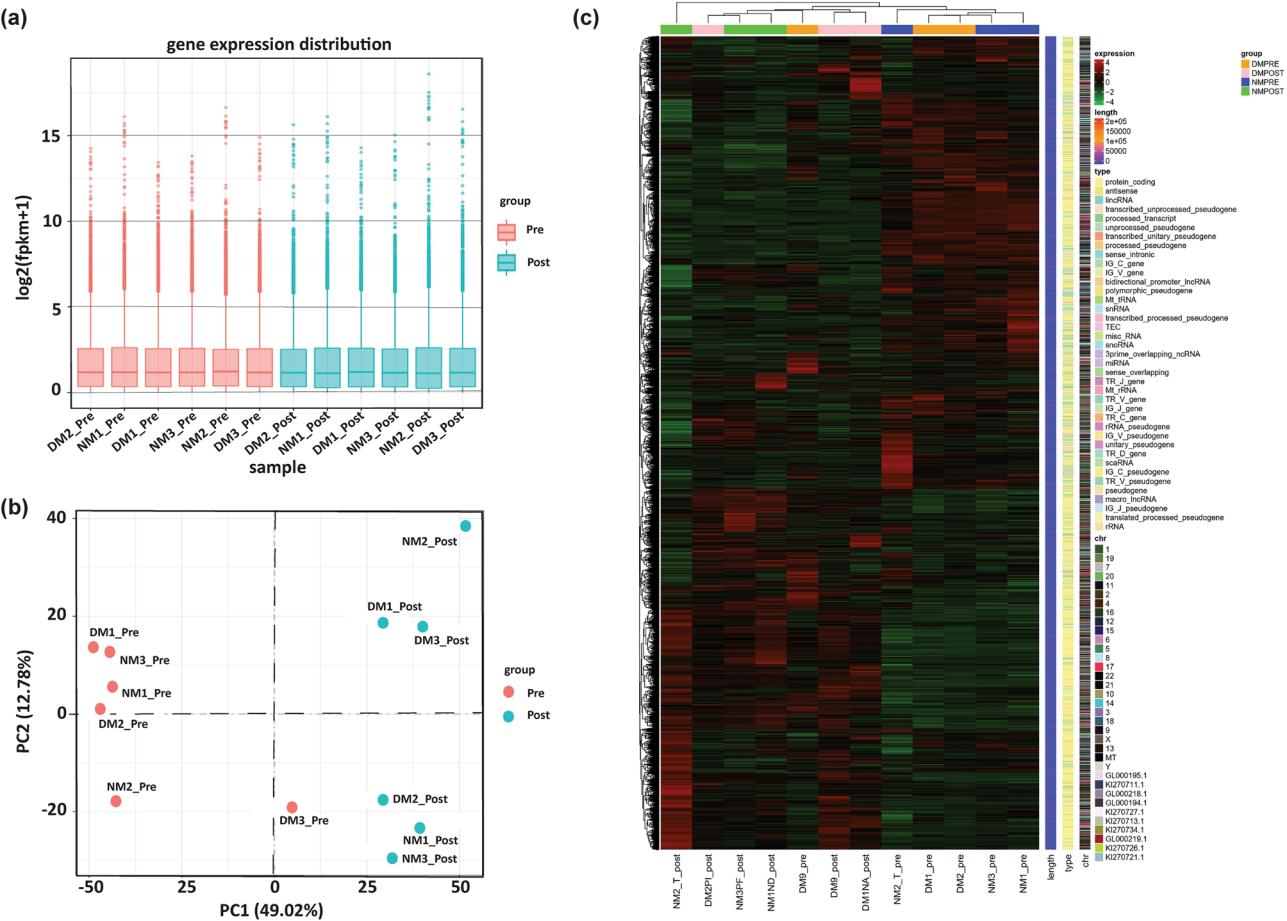
Transcriptomic analysis. (**a**) A box plot displaying the mRNA expression levels (FPKM) distribution for each sample. The horizontal axis represents the sample name, while the vertical axis represents log_2_(FPKM + 1). (**b**) Principal Component Analysis (PCA) plot illustrating distinctions between different experimental groups. The horizontal (PC1) and vertical axes (PC2) depict the two most significant principal components. PCA delineated the pre-treatment group, NM-post, and DM-post into three distinct cluster regions. (**c**) Transcriptomic heatmap depicting hierarchical clustering of all differentially expressed genes (DEGs). The Log_2_FPKM value was used for clustering, with high-expression genes in red and low-expression genes in green. The horizontal axis depicts the sample, while the vertical axis denotes the differential gene. Genes are clustered on the left based on expression similarity, and each sample is clustered at the top based on expression profile similarity. The color spectrum from green to red signifies escalating gene expression levels. FPKM, Fragments Per Kilobase of transcript per Million mapped reads; PC, principal component; NM-post, IronQ-treated PBMCs from healthy donors; DM-post, IronQ-treated PBMCs from diabetes mellitus donors.

RNA sequencing (RNA-Seq) was utilized to examine and describe alterations in gene expression patterns resulting from exposure to IronQ for a duration of 10 days in peripheral blood mononuclear cells (PBMCs) obtained from three persons diagnosed with diabetes mellitus (DM) and three age-matched healthy individuals serving as a control group. Over 81 million clean sequence reads were obtained on average across 12 samples, and the depth, quality, and alignment rates of the sequencing are displayed in Supplementary Table S1. The 12 samples shared a reasonably uniform distribution of gene expression levels, as shown by the box plot of the distribution of mRNA expression levels with respect to Fragments Per Kilobase of transcript per Million mapped reads (FPKM) among the four groups (Fig. 1a). As illustrated in Fig. 1b, the principal component analysis (PCA) plot revealed that in PC1, the pre-treatment of the healthy and DM samples was clustered together, indicating similar mRNA profiles between the pre-treatment groups. However, the mRNA profiles of the IronQ post-treatment group differed significantly from the respective pre-treatment controls in both the healthy and DM samples. Additionally, it was demonstrated that there were some differences between the two post-treatment groups from the healthy samples and DM samples in PC2. By using an unsupervised hierarchical clustering approach, we created heatmaps to reveal how the expression profiles of differentially expressed genes (DEGs) differed among the groups (Fig. 1c). It was observed that the samples from the four groups exhibited clustering tendencies when subjected to pairwise comparisons. The RNA-seq results mentioned above indicated that the DEG data were reliable and that the mRNA profiles of the IronQ post-treatment group were different from those of the pre-treatment group.

### Differentially expressed genes in IronQ-treated peripheral blood mononuclear cells (PBMCs) from healthy donors and related functional analysis

**Figure 2:**
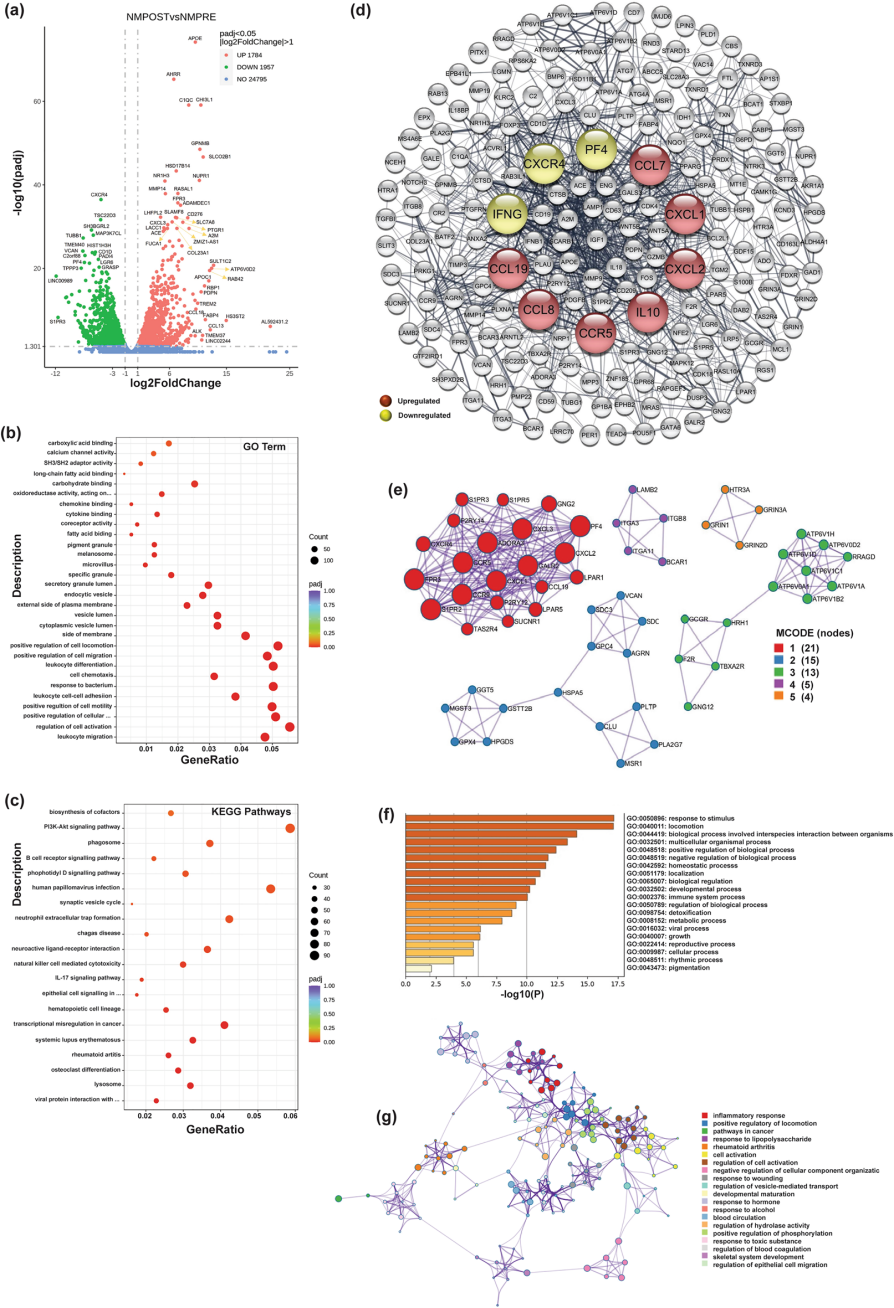
The effects of IronQ on peripheral blood mononuclear cells (PBMCs) from healthy donors. (**a**) Volcano plot displaying the differentially expressed genes (DEGs) of IronQ-treated PBMCs (NM-post) vs. untreated PBMCs (NM-pre) from healthy donors, analyzed by DESeq2 (|log_2_ FC|≥ 1; adj p-value ≤ 0.05). The horizontal axis represents the log_2_ Fold Change of gene expression, where larger absolute values indicate more incredible fold changes between samples. The vertical axis represents the statistical significance of the expression difference. Green dots signify down-regulated DEGs; red dots indicate upregulated DEGs, and blue dots represent detected but not significantly differentially expressed genes. (**b**) Top 20 gene ontology (GO) enrichment analyses and (**c**) Top 20 KEGG enrichment analyses of the DEGs between NM-post vs. NM-pre. The horizontal axis represents the gene ratio, which is the ratio of DEGs annotated in the GO term/ KEGG pathways to the total number of genes in the reference data set. The vertical axis represents the GO term/ KEGG pathways. The dot color indicates the adjusted p-value, with lower values indicating more significant enrichment. The dot size reflects the number of genes enriched in the corresponding GO term/ KEGG pathways. (**d**) Protein–protein interaction (PPI) network analysis focusing on the top 200 DEGs (ranked by P-value). The top 10 ranked hub genes of PPI networks were obtained from CytoHubba analysis and are highlighted in red (upregulated genes) and yellow (downregulated genes). (**e**) MCODE clustering analysis of PPI networks using Cytoscape software, representing the first 5 functional clusters of the PPI network. (**f**) Bar plot illustrating GO functional and KEGG pathway enrichment of hub genes, showing the enriched terms of hub genes in response to IronQ treatment of healthy PBMCs. The vertical axis represents the significantly enriched terms, each bar describing the number of mapped annotated genes in the reference data set. The horizontal axis indicates each category's significance (− log_10_ p-value). (**g**) Network of enrichment term analysis across the hub genes in response to IronQ treatment of healthy PBMCs.

Initially, we examined the gene expression shifts between IronQ-treated PBMCs from healthy donors (NM-post) and untreated PBMCs (NM-pre). The volcano plot analysis highlighted a notable alteration in the expression of 3741 genes, meeting the criteria of |log2 fold change |≥ 1 and adjusted *p*-value (padj) of less than 0.05. Among these, 1784 genes (47.7%) showed upregulation, while 1957 genes (52.3%) displayed downregulation in NM-post compared to NM-pre, as illustrated in Fig. 2a.

To comprehensively understand the impact of IronQ on PBMCs, we conducted a Gene Ontology (GO) enrichment analysis that covered three GO categories: biological processes (BPs), cellular components (CCs), and molecular functions (MFs). This analysis aimed to pinpoint crucial biological processes and pathways affected when PBMCs interacted with IronQ. The treatment of healthy PBMCs with IronQ resulted in a notable enrichment of 390 GO terms (*p* < 0.05), which encompassed 351 GO terms related to biological processes (BPs), 27 GO terms related to cellular components (CCs), and 12 GO terms related to molecular functions (MFs) (sheet 1 of Supplementary Table S2). Furthermore, to emphasize the extensive impact of IronQ on PBMCs, we highlighted important GO terms with high significance (the lowest P-value) in each category. The findings, which are presented in Fig. 2b, demonstrated that biological processes related to leukocyte migration, the regulation of cell activation, and the positive regulation of cellular component movement were primary biological responses to IronQ exposure. In the cellular component category, the GO terms that exhibited a notable enrichment included the cytoplasmic vesicle lumen, endocytic vesicle, secretory function, focal adhesion, and lysosomal membrane. The DEGs encompassed a spectrum of molecular functions such as binding to fatty acids, coreceptor activity, cytokine binding, chemokine binding, oxidoreductase activity, and calcium channel activity (the complete information for the significantly enriched GO terms of DEGs up- and downregulated genes among the DEGs is shown in sheets 2 and 3 of Supplementary Table S2, respectively).

Our pathway analysis using Kyoto Encyclopedia of Genes and Genomes (KEGG) annotations identified the top twenty enriched pathways. This significant finding includes lysosome, osteoclast differentiation, rheumatoid arthritis, and IL17 signaling pathways (Fig. 2c and sheet 4 of Supplementary Table S2). The KEGG enrichments found in the DEGs, explicitly focusing on up- and downregulated genes are visually represented in Supplementary Fig. S1a,b, respectively (for a comprehensive view, refer to sheets 5 and 6 of Supplementary Table S2).

The alterations, a key aspect of our study, induced by IronQ in healthy peripheral blood mononuclear cells (PBMCs) prompted us to investigate specific regulatory pathways. Thus, we thoroughly selected the top 200 differentially expressed genes (DEGs) based on their p-values (see sheet 1 of supplementary Table S3). The expression patterns of these DEGs were visualized through a heatmap, as showcased in Figure S2. Recognizing the pivotal role of protein interactions in determining protein function, we further explored the functional relationships among the identified 200 DEGs by constructing a protein–protein interaction (PPI) network. Notably, most genes exhibited interactions with one another, with only 12 genes lacking such connections. To further understand the functional significance of the PPI network, we employed the advanced cytoHubba plugin in Cytoscape. This tool helped us identify hub genes, which are highly connected nodes within the PPI network. Our analysis revealed the top 10 hub genes, namely CCL19, CCL7, CCL8, CCR5, CXCR4, CXCL1, CXCL2, IL10, PF4, and IFNG (Fig. 2d). These hub genes are crucial in the context of IronQ treatment. Furthermore, we utilized the MCODE plugin to uncover highly interconnected regions, referred to as clusters, within the PPI network. The analysis revealed five distinct MCODE clusters with notable functional enrichments. Cluster 1, comprising 21 nodes and 163 edges, was highly enriched in the chemokine-mediated signaling pathway. Cluster 2, with 15 nodes and 30 edges, is primarily associated with glutathione metabolism. Cluster 3 displayed significant enrichment in synaptic vesicle lumen acidification, featuring 13 nodes and 36 interactions. Cluster 4, encompassing 5 nodes and 9 edges, exhibited gene enrichment primarily in the integrin-mediated signaling pathway and focal adhesion. Finally, cluster 5, consisting of 4 nodes and 6 interactions, showed notable enrichment in the ligand-gated ion channel signaling pathway (Fig. 2e). Functional and biological process enrichment analyses of the hub genes in response to IronQ treatment of healthy PBMCs were conducted by using Metascape. The results demonstrated that the identified genes were primarily associated with inflammatory response, positive regulation of locomotion, cell activation, and detoxification (Fig. 2f). The network diagram visually represents the interconnectedness of the most enriched terms (Fig. 2g).

The analysis highlights a significant enrichment of DEGs in diverse biological processes. These include chemotaxis, locomotion, response to stimuli, pathways in cancer, angiogenesis, and the immune system. These findings suggest that these hub genes, influenced by IronQ treatment, play pivotal roles in governing the migration, differentiation, proliferation, immunoregulation, and regeneration of healthy PBMCs.

### Differentially expressed genes in IronQ-treated peripheral blood mononuclear cells (PBMCs) of diabetic donors and related functional analysis

**Figure 3:**
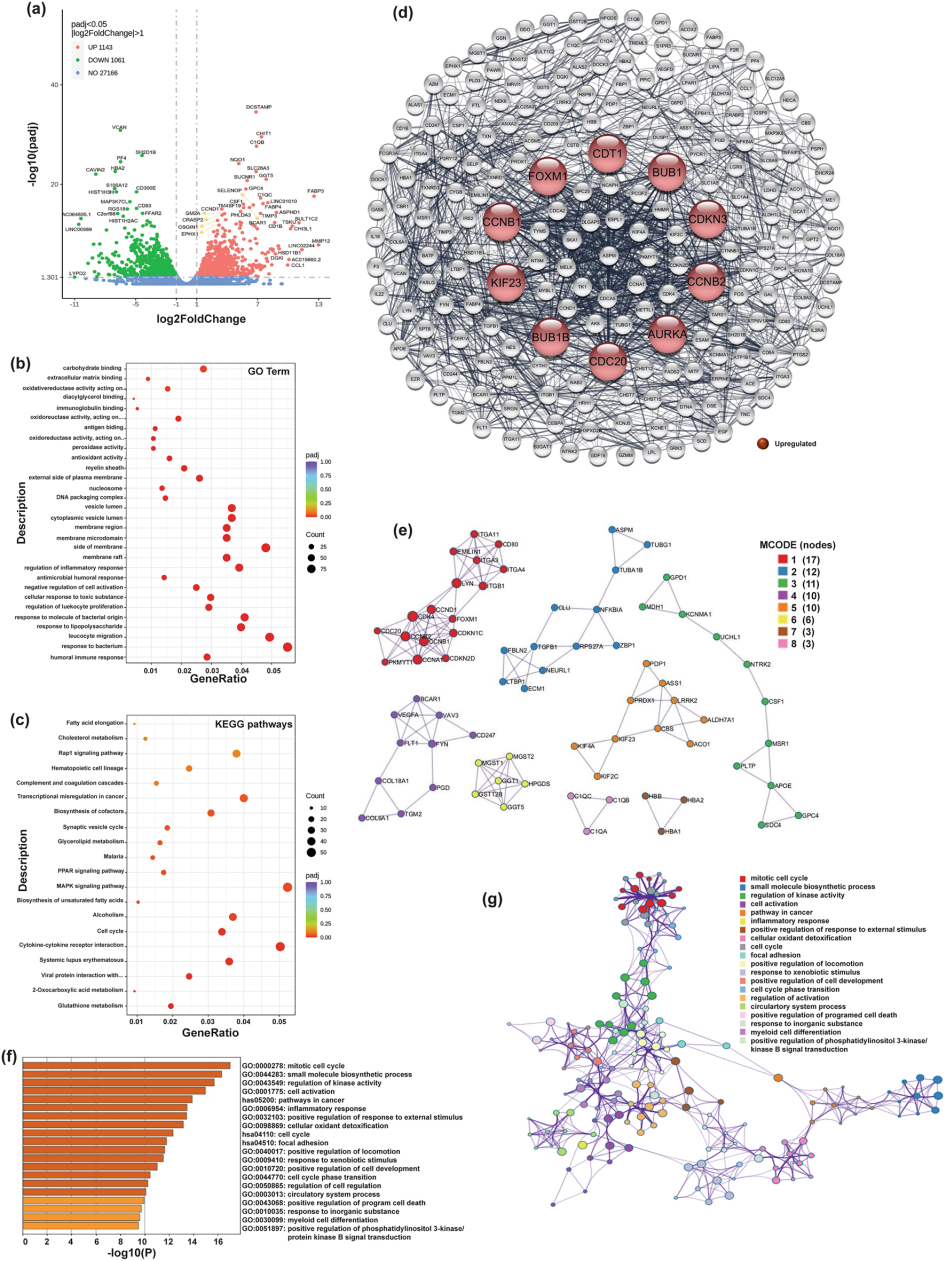
The effects of IronQ on peripheral blood mononuclear cells (PBMCs) from diabetes donors. (**a**) Volcano plot displaying the differentially expressed genes (DEGs) of IronQ-treated PBMCs (DM-post) vs. untreated PBMCs (DM-pre) from diabetes donors, analyzed by DESeq2 (|log_2_ FC|≥ 1; adj p-value ≤ 0.05). The horizontal axis represents the log_2_ Fold Change of gene expression, where larger absolute values indicate more incredible fold changes between samples. The vertical axis represents the statistical significance of the expression difference. Green dots signify down-regulated DEGs; red dots indicate upregulated DEGs, and blue dots represent detected but not significantly differentially expressed genes. (**b**) Top 20 gene ontology (GO) enrichment analyses and (**c**) Top 20 KEGG enrichment analyses of the DEGs between DM-post vs. DM-pre. The horizontal axis represents the gene ratio, which is the ratio of DEGs annotated in the GO term/ KEGG pathways to the total number of genes in the reference data set. The vertical axis represents the GO term/ KEGG pathways. The dot color indicates the adjusted p-value, with lower values indicating more significant enrichment. The dot size reflects the number of genes enriched in the corresponding GO term/ KEGG pathways. (**d**) Protein–protein interaction (PPI) network analysis focusing on the top 200 DEGs (ranked by P-value). The top 10 ranked hub genes of PPI networks were obtained from CytoHubba analysis and are highlighted in red (upregulated genes). (**e**) MCODE clustering analysis of PPI networks using Cytoscape software, representing the first 5 functional clusters of the PPI network. (**f**) Bar plot illustrating GO functional and KEGG pathway enrichment of hub genes in response to IronQ treatment of diabetes PBMCs. The vertical axis represents the significantly enriched terms, each bar describing the number of mapped annotated genes in the reference data set. The horizontal axis indicates each category's significance (-log_10_ p-value). (**g**) Network of enrichment term analysis across the hub genes in response to IronQ treatment of diabetes PBMCs.

Next, we analyzed and compared the gene expression patterns in IronQ-treated PBMCs obtained from donors with diabetes (referred to as DM-post) with those whose PBMCs remained untreated (referred to as DM-pre). The volcano plot in Fig. 3a graphically illustrates a significant alteration in the expression of 2204 genes, meeting the criteria of |log2 fold change|≥ 1 and adjusted *p*-value (padj) less than 0.05, as revealed by our analysis of the DEGs. Within this gene set, 1143 (51.9%) exhibited upregulated expression, while 1061 (48.1%) showed downregulated expression in the DM-post group compared to the DM-pre group.

After identifying 2204 DEGs, we performed a DEG enrichment analysis by utilizing functional annotations from the GO and KEGG databases. This analysis revealed significant enrichment in 424 GO terms (371 GO terms related to biological processes, 29 GO terms related to cellular compartments, and 24 GO terms related to molecular functions). These enriched GO terms spanned various biological processes, including regulation of inflammatory response, wound healing, cellular detoxification, and negative regulation of leukocyte activation. Detailed information on these significantly enriched GO terms is provided on Sheet 1 of Supplementary Table S4. Figure 3b presents the top 20 GO terms that exhibited notable enrichment. Additionally, the upregulated genes showed substantial enrichment in processes related to cellular oxidant detoxification and lipid catabolic processes, as illustrated in Supplementary Fig. S3a and detailed in Sheet 2 of Supplementary Table S4. Conversely, the enrichment analysis of downregulated genes within the DEG dataset was associated with processes related to the response to a bacterium, T cell activation, and antibacterial humoral response, as depicted in Supplementary Fig. S3b and outlined in Sheet 3 of Supplementary Table S3. The KEGG pathway analysis unveiled 16 prominently enriched pathways, as displayed in Fig. 3c and detailed in Supplementary Table S4 on sheet 4. These pathways encompass a range of biological processes, including glutathione metabolism, 2-oxocarboxylic acid metabolism, systemic lupus erythematosus, the cell cycle, the MAPK signaling pathway, and the PPAR signaling pathway. Among these pathways, the upregulated KEGG pathways were notably enriched in glycerolipid metabolism, glutathione metabolism, and the cell cycle, while the downregulated genes displayed enrichment in pathways related to systemic lupus erythematosus and neutrophil extracellular trap formation. For comprehensive details concerning the significantly enriched KEGG pathways of DEGs for both upregulated and downregulated genes, please refer to sheets 5 and 6, respectively, of Supplementary Table S4.

To further explore the specific biological pathway involved in IronQ treatment in diabetes PBMCs. We identified the top 200 DEGs (ranked by p-value) that exhibited significant alterations after IronQ treatment. These are crucial in understanding the specific biological pathways involved in IronQ treatment in diabetes PBMCs. A comprehensive list of these top 200 DEGs is provided on sheet 2 of Supplementary Table S3, and their expression patterns are graphically illustrated in the heatmap shown in Supplementary Fig. S4. To visualize the interactions among these DEGs, we constructed a PPI network, as depicted in Fig. 3d. Most of these genes exhibited intricate interactions with each other, and only nine genes lacked any such interactions. Furthermore, we utilized the cytoHubba plugin in Cytoscape to identify hub genes, which represent highly connected nodes within the PPI network. Particularly noteworthy were the top 10 hub genes exhibiting the highest connectivity degree. These hub genes included *AURKA, CCNB1, CCNB2, CDC20, CDKN3, KIF23, CDT1, BUB1, BUB1B,* and *FOXM1* (see Fig. 3d). These genes are closely associated with the effects of IronQ treatment on diabetes PBMCs, highlighting their potential significance in mediating the observed biological responses. Moreover, we employed the MCODE plugin to investigate densely interconnected regions that were recognized as clusters within the PPI network. This enabled us to gain a deeper understanding of the relationships between the genes forming the network. Figure 3e shows the eight MCODE clusters characterized by the most elevated scores. Cluster 1, which comprised 17 nodes and 59 edges, exhibited significant enrichment in the cell cycle. Cluster 2, which involved 12 nodes and 17 edges, was primarily associated with the inflammatory response. Furthermore, Cluster 3, which featured 11 nodes and 13 edges, displayed significant enrichment in sterol transport. Cluster 4, which encompassed 10 nodes and 18 edges, showed genes that were primarily enriched in the vascular endothelial growth factor receptor signaling pathway and focal adhesion. Additionally, Cluster 5, which covered 10 nodes and 15 edges, exhibited notable enrichment in the biosynthesis of amino acids. Glutathione metabolism was significantly enriched for cluster 6, which involved 6 nodes and 15 edges. Cluster 7, with three nodes and three interactions, was mainly associated with oxygen and nitrogen transport. Lastly, Cluster 8 showed significant enrichment in cell function disassembly, featuring three nodes and three edges.

To understand the cellular processes related to the genes, functional and biological process enrichment analyses of the hub genes in response to IronQ treatment of diabetes PBMCs were conducted by using Metascape. We found that the genes were enriched in several GO terms and KEGG pathways, as depicted in Fig. 3f. These enrichments were primarily associated with the cell cycle, cellular oxidant detoxification, focal adhesion, and positive regulation of cell development. The network diagram depicted in Fig. 3g provides a visual representation that depicts the intricate interconnections and associations among the highly enriched terms derived from the analysis.

These findings strongly suggest that these cluster genes may play pivotal regulatory roles in fundamental biological processes, including the precise orchestration of the cell cycle, active cell proliferation, cell development, and precise immunoregulation. This underscores the potential significance of these genes in mediating the biological responses to IronQ treatment in DM-PBMCs.

### Common differentially expressed genes in healthy and diabetic peripheral blood mononuclear cells under IronQ treatment conditions

**Figure 4:**
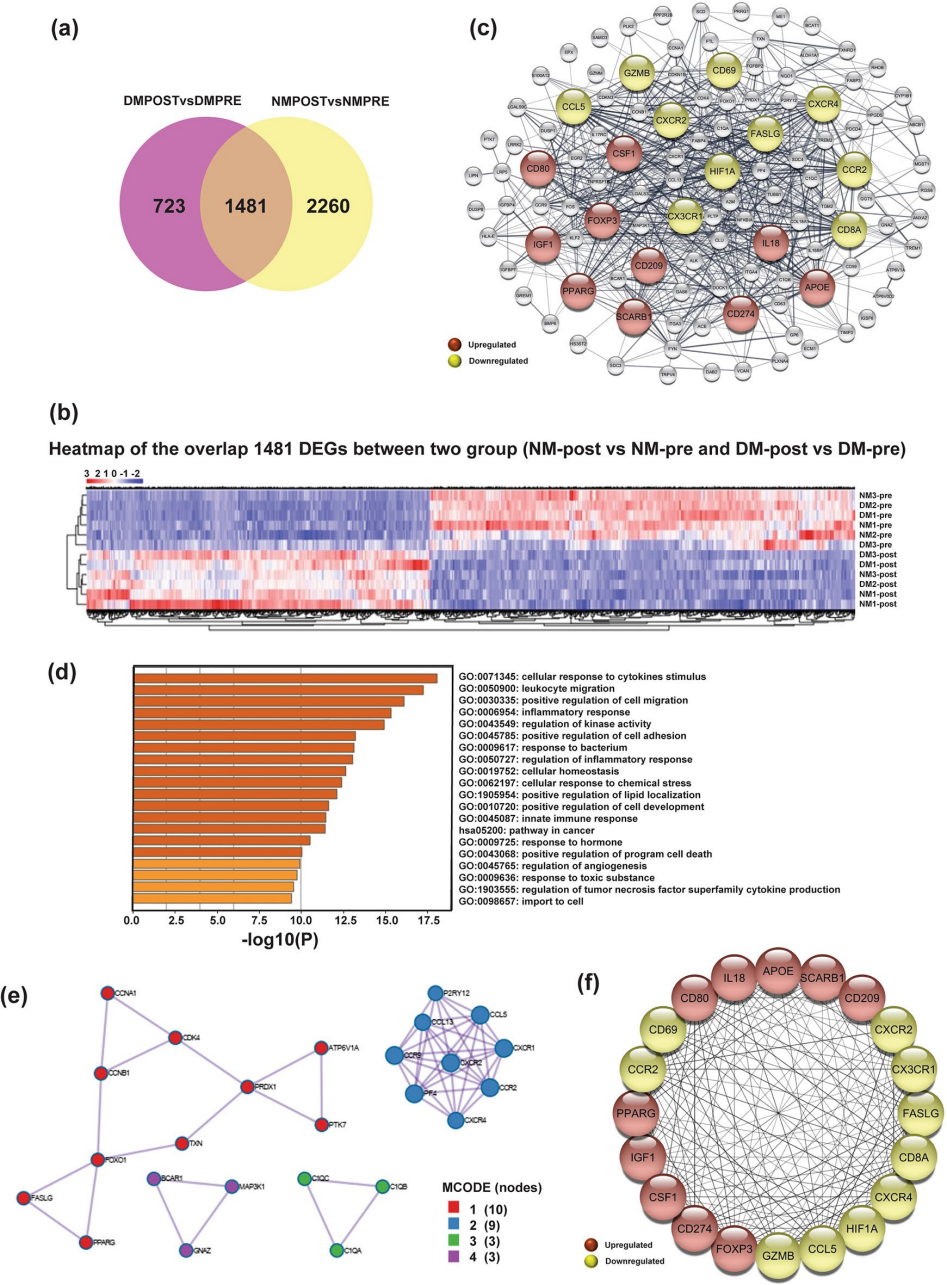
The common effects of IronQ on both healthy and diabetic peripheral blood mononuclear cells (PBMCs). (**a**) Venn diagram displaying the overlap of differentially expressed genes (DEGs), revealing 1481 common DEGs between the two groups NM-post vs. NM-pre and DM-post vs. DM-pre comparisons. (**b**) Heatmap representing the expression patterns of the 1481 DEGs common to both groups, with 662 genes upregulated and 819 genes downregulated. Genes are clustered at the bottom based on expression similarity, while each sample is clustered on the left based on profile similarity. (**c**) Protein–protein interaction (PPI) network was constructed using the top 120 DEGs, ranked by their p-values. The top 20 ranked hub genes of PPI networks, identified through CytoHubba analysis, are highlighted in red (upregulated genes) and yellow (downregulated genes). (**d**) Bar plot illustrating Gene Ontology (GO) and Kyoto Encyclopedia of Genes and Genomes (KEGG) pathway enrichment of hub genes common in response to IronQ treatment of PBMCs. (**e**) MCODE clustering analysis of PPI networks using Cytoscape software, representing the top 4 functional clusters of the PPI network. (**f**) The PPI network comprises the top 20 hub genes, with upregulated genes marked in red and downregulated genes highlighted in yellow, representing their protein–protein interaction degree. NM-post, IronQ-treated PBMCs from healthy donors; NM-pre, untreated PBMCs from healthy donors; DM-post, IronQ-treated PBMCs from diabetes mellitus donors; DM-pre, untreated PBMCs from diabetes mellitus donors.

After the IronQ treatment, 1481 DEGs were commonly pinpointed in healthy and diabetic PBMCs (Fig. 4a). This set encompassed 662 upregulated and 819 downregulated DEGs (Fig. 4b). Furthermore, it was notable that the number of downregulated genes exceeded that of the upregulated genes. The common DEGs displayed significant enrichments in various biological processes associated with immune system function, response to stimuli, cell activation, cell migration, cell population proliferation, small-molecule metabolism, and regulation of developmental processes (Supplementary Fig. S5a). Moreover, 11 KEGG pathways exhibited notable enrichments; these included systemic lupus erythematosus, glutathione metabolism, metabolic pathways, phagosomes, and biosynthesis of amino acids (Supplementary Fig. S5b). Additionally, the upregulated genes showed substantial enrichments in pathways related to glutathione metabolism, the PPAR signaling pathway, and lysosomes. However, the enrichment analysis of downregulated genes was associated with pathways related to systemic lupus erythematosus, neutrophil extracellular trap formation, and natural-killer-cell-mediated cytotoxicity, as depicted in Supplementary Fig. S5c,d, respectively.

Among the common 1481 DEGs, we carefully selected the top 120 DEGs, ranked by p-value, which exhibited the most pronounced effects from IronQ treatment in both PBMCs from healthy individuals and those with diabetes. These selected genes were utilized for constructing a PPI network analysis, as illustrated in Fig. 4c. Furthermore, to thoroughly explore genes potentially playing crucial roles in the context of IronQ treatment in PBMCs, we identified hub genes from this network using the cytoHubba plug-in in Cytoscape. This analysis identified 20 hub genes, comprising ten upregulated DEGs and ten downregulated DEGs. Among the upregulated DEGs were *IL18, CD80, FOXP3, CSF-1, IGF-1, CD209, CD274, PPARG, SCARB1*, and *APOE*. Conversely, the downregulated DEGs included *HIF1A, FASLG, GZMB, CCL5, CX3CR1, CCR2, CXCR4, CXCR2, CD69*, and *CD8A*. We further identify interconnected clusters within the PPI network using the MCODE plugin. This analysis unveiled three distinct modules characterized by specific biological processes and pathways. The first cluster was associated with cellular senescence and pathways in cancer, indicating potential involvement in cellular aging processes and cancer-related pathways. The second cluster was linked to the cytokine-mediated signaling pathway, suggesting a role in immune response regulation and inflammatory processes. The third cluster was associated with cell junction disintegration, indicating potential involvement in cell adhesion and migration processes (Fig. 4e). Of particular interest, we observed intricate interconnections among 20 hub genes in additional PPI network studies, as depicted in Fig. 4f. This underscores the complexity of interactions among the identified hub genes and highlights their potential roles in mediating biological processes related to IronQ treatment in PBMCs and are thus of particular interest for further investigation.

An enrichment analysis of biological pathways was conducted with the hub genes. Many pivotal biological processes and pathways were elucidated within this network, and they included crucial aspects such as the inflammatory response, cell migration, cellular response to stress, and angiogenesis. Furthermore, the notably enriched pathways associated with upregulated genes included pathways in cancer and regulation of angiogenesis (Fig. 4d). The convergence of these pathways, together with the significant upregulation of critical cytokines and growth factor genes, such as *IL18*, *CSF-1*, and *IGF-1*, among others, strongly suggested a transcriptomic profile in IronQ-treated PBMCs that aligned with repair processes. Hence, it is reasonable to deduce that, whether applied to healthy or diabetic PBMCs, IronQ treatment consistently triggered the cellular immune response and exerted discernible immunomodulatory effects. Additionally, the stimulated cells underwent metabolic shifts that were conducive to enhancing their reparative properties.

### Unique differentially expressed genes of healthy and diabetic peripheral blood mononuclear cells under IronQ treatment conditions

One of the primary objectives of this study was to examine the regenerative potential of PBMCs obtained from healthy individuals and those with diabetes mellitus under IronQ treatment conditions and to explore their suitability for autologous cell therapy. As observed in our study, IronQ treatment consistently elicited cellular immune responses and exerted discernible immunomodulatory effects in PBMCs, irrespective of whether they were obtained from healthy or diabetic individuals, as mentioned above. However, it is noteworthy that under the IronQ treatment conditions, PBMCs from normal and diabetic individuals exhibited differences in their mode of cell–cell signaling. This indicates potential variations in cellular responses to IronQ treatment between individuals with and without diabetes, which may have implications for the efficacy of autologous cell therapy in diabetic patients.

**Figure 5:**
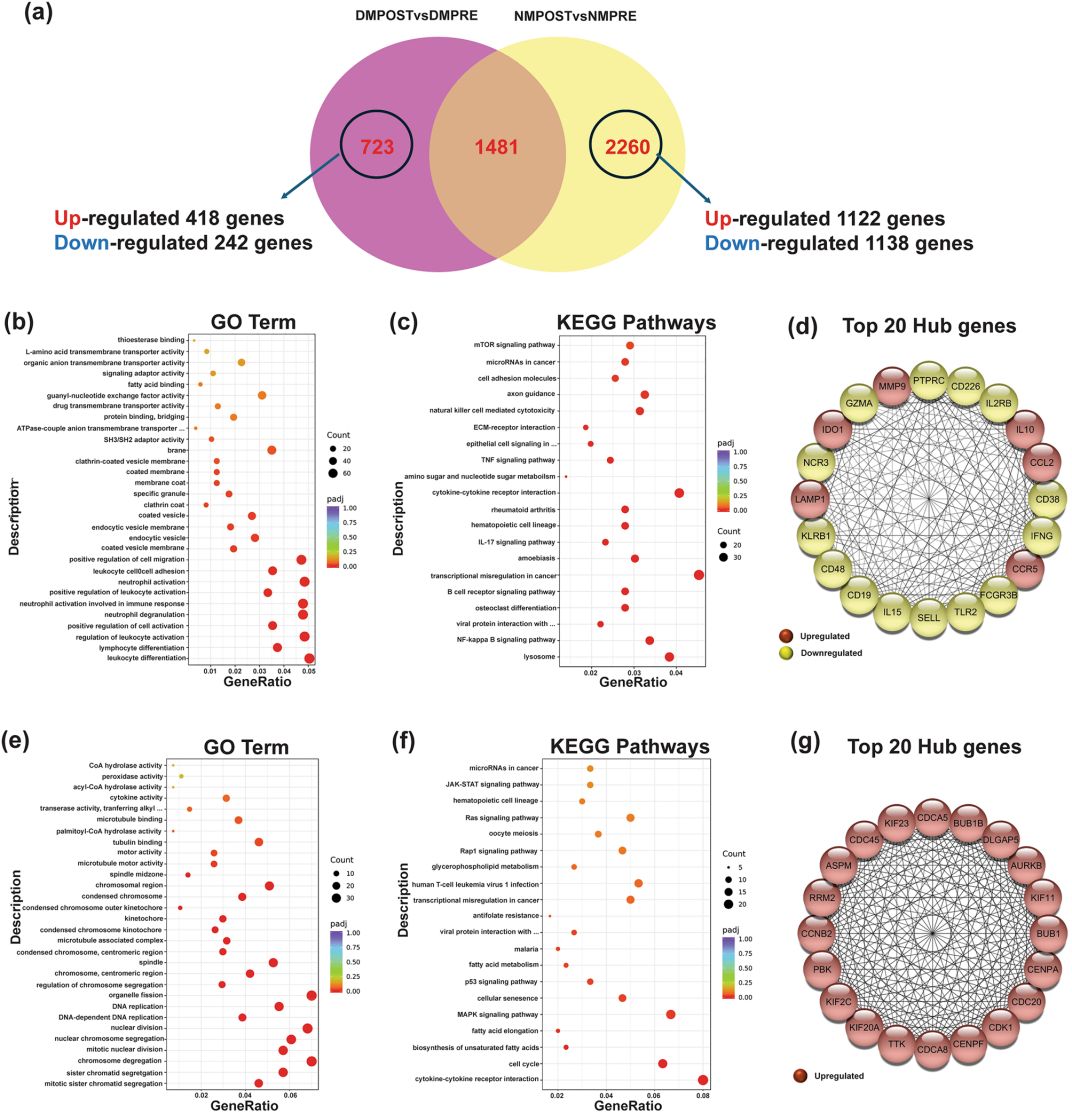
The unique effects of IronQ on healthy and diabetic peripheral blood mononuclear cells (PBMCs). (**a**) Venn diagram of the DEGs showing 2260 unique DEGs in healthy donor PBMCs and 723 unique DEGs in diabetes mellitus donor PBMCs. (**b**) Gene Ontology (GO) enrichment analysis and (**c**) Kyoto Encyclopedia of Genes and Genomes (KEGG) enrichment analysis of unique DEGs in healthy donor PBMCs. (**d**) The PPI network of the top 20 hub genes among the unique DEGs in healthy donor PBMCs, representing their protein–protein interaction degree, with upregulated genes being marked in red and downregulated genes being highlighted in yellow. (**e**) Gene Ontology (GO) enrichment analysis and (**f**) Kyoto Encyclopedia of Genes and Genomes (KEGG) enrichment analysis of unique DEGs in diabetic donor PBMCs. The horizontal axis represents the gene ratio, which is the ratio of DEGs annotated in the GO term/ KEGG pathways to the total number of genes in the reference data set. The vertical axis represents the GO term/KEGG pathways. The dot color indicates the adjusted p-value, with lower values indicating more significant enrichment. The dot size reflects the number of genes enriched in the corresponding GO term/ KEGG pathways. (**g**) The PPI network of the top 20 hub genes among the unique DEGs in diabetes mellitus donor PBMCs represents their protein–protein interaction degree, with upregulated genes marked in red.

To ascertain the disparate effects of IronQ on healthy and DM PBMCs, we attempted to conduct a comprehensive exploration that involved identifying specific genes that were differentially expressed in response to IronQ treatment in healthy and DM PBMCs. Furthermore, we intended to elucidate the different processes and pathways associated with the impact of IronQ by using PPI analysis and KEGG enrichment analysis. We found that the PBMCs from healthy donors exhibited a notably heightened expression of unique genes in comparison with those from DM donors. A significant quantity of distinct DEGs were discernible in the PBMCs of healthy subjects after the IronQ treatment (NM-post). This cohort of DEGs encompassed 2260 genes, with 1122 demonstrating upregulation and 1138 displaying downregulation. Conversely, the impact of IronQ on the PBMCs from DM donors (DM-post) revealed a comparatively diminished magnitude. In this specific context, a total of 723 genes were identified. Among these genes, 481 exhibited upregulation, while 242 were downregulated in comparison with the untreated controls (Fig. 5a).

For the healthy PBMCs, the treatment of IronQ exhibited a discernible impact on the DEGs that were enriched in many biological processes and pathways. These encompassed processes related to endocytosis, cell chemotaxis, regulation of cell migration, and lymphocyte differentiation (Fig. 5b). Moreover, distinct pathways exhibited significant enrichment in healthy PBMCs after the IronQ treatment; these notably included the lysosome, IL17 signaling pathway, NF-kappa B signaling pathway, axon guidance, mTOR signaling pathway, B cell receptor signaling pathway, and natural-killer-cell-mediated cytotoxicity (Fig. 5c). Following this, cytoHubba analysis successfully revealed 20 hub genes from a pool of 2260 distinct DEGs (Fig. 5d). Among them, six DEGs displayed upregulation—namely, *CCR5*, *IL10*, *CCL2*, *LAMP1*, *MMP9*, and *IDO*. These upregulated hub genes were associated with angiogenesis and the immunomodulatory milieu. Conversely, the analysis revealed fourteen downregulated DEGs: *IGFN*, *FCGR3B*, *CD19*, *PTPRC*, *IL15*, *SELL*, *CD38*, *GZMA*, *IL2RB*, *NCR3*, *KLRB1*, *CD226*, *CD48*, and *TLR2*. Notably, these fourteen downregulated hub genes were predominantly enriched and actively involved in critical processes associated with inflammatory response, including neutrophil-mediated immunity, T cell differentiation, T cell activation, and cell adhesion molecules.

On the contrary, we observed a significant enrichment of cell cycle regulation, the p53 signaling pathway, and the MAPK signaling pathway derived from diabetes donors due to the IronQ treatment in PBMCs (Fig. 5e,f). In the PPI network, a total of 723 genes were filtered; the top 20 hub genes with the highest degrees were identified, namely, *RRM2, KIF11, BUB1B, KIF2C, CDCA8, PBK, CDC45, KIF23, CDK1, KIF20A, BUB1, AURKB, TTK, DLGAP5, ASPM, CENPA, CENPF, CDC20, CCNB2*, and *CDCA5* (Fig. 5g). Notably, these 20 specific genes showed enrichments in cell-cycle-related terms, including such processes as cell division, spindle organization, nuclear division, the mitotic cell cycle checkpoint, and organelle fission. A noteworthy observation was made regarding DEGs in a smaller module, revealing a relationship with cholesterol metabolism. Specifically, genes such as *CCL17, CD163, APOE, CYP27A1*, and *STAB1*, some of which are recognized as anti-atherogenic and anti-inflammatory factors in diabetes patients, were identified within this module.

### Validation of candidate genes of healthy and diabetic peripheral blood mononuclear cells under IronQ treatment conditions

**Figure 6:**
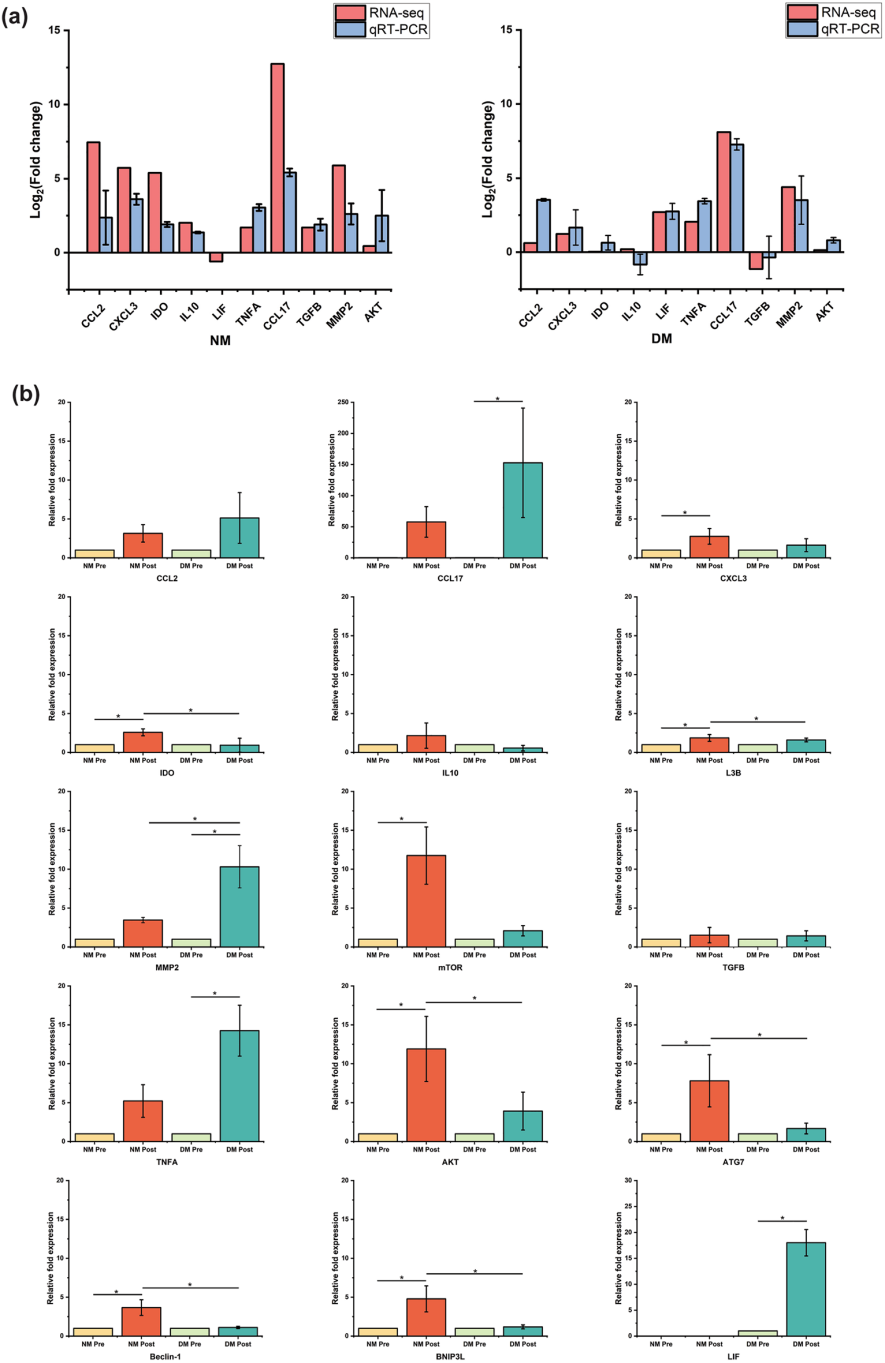
Validation of the RNA-sequencing analysis with quantitative real-time PCR (qRT-PCR). (**a**) Comparison plot displaying the Log_2_ fold change values for 10 candidate genes obtained from both RNA-Seq and the qRT-PCR analysis. (**b**) qRT-PCR results for 15 selected genes showing the relative fold change of each condition (NM: PBMCs from healthy donors; DM: PBMCs from diabetic donors). Statistical significance was assessed through ANOVA followed by Tukey's post-hoc test. In the plots, the presence of (*) denotes statistically significant differences, signifying a significance level of *p* < 0.05.

Subsequently, we performed qRT-PCR to validate the gene expression changes identified through the RNA-seq analysis. We selected 10 DEGs that were associated with immunomodulation, angiogenesis, and wound healing for a focused validation across all experimental groups. The results in Fig. 6a demonstrate a high level of concordance between the qRT-PCR and RNA-seq data. Specifically, we observed the upregulation of nine genes and the downregulation of one gene (*LIF*) following the IronQ treatment in healthy PBMCs. In the case of DM PBMCs, our results revealed a consistent elevation in the expression of nine genes and the downregulation of one gene (*TGFB*), confirming the findings from RNA sequencing. The consistency in expression patterns between RNA-seq and qRT-PCR further validated our results. We conducted a comparative analysis across the four experimental conditions to assess the mRNA expression levels of PBMC genes linked to immunomodulation, tissue repair, and autophagy in response to the IronQ treatment, as demonstrated in Fig. 6b. We found a notable impact of the IronQ treatment on the expression of *CXCL3*, *IDO*, *LC3B*, *Atg7*, *Beclin1*, and *Bnip3L* in comparison with the control group that did not undergo any treatment. DM PBMCs treated with IronQ showed a substantial change in the mRNA expression of *TNFA*, *LIF*, *CCL17*, and *MMP2* in comparison with that in the untreated control group. Remarkably, the expression of *CCL17* mRNA, a notable M2 marker, was significantly impacted by the IronQ treatment in PBMCs from individuals with diabetes. Notably, the upregulation of *CCL17* expression in diabetic PBMCs was approximately double compared to that in PBMCs from healthy individuals. Conversely, in healthy PBMCs, we observed a significant upregulation of *IDO* with respect to the DM PBMCs after the treatment with IronQ. Furthermore, the levels of mRNA expression related to autophagic markers such as *Bnip3L*, *LC3B*, *beclin1*, and *Atg7* were notably higher in the IronQ-treated healthy PBMCs than in those from DM donors. These findings suggest that in addition to its immunomodulatory and tissue repair properties, IronQ influences the autophagic pathway in PBMCs of healthy individuals, potentially enhancing their robustness to survival challenges during stress conditions. Conversely, for DM PBMCs, which are characterized by chronic inflammation, IronQ stimulates the polarization of M2 macrophages, creating an anti-inflammatory environment.

### Characterizing cellular phenotypes in peripheral blood mononuclear cells of healthy and diabetic donors under IronQ treatment conditions

**Figure 7:**
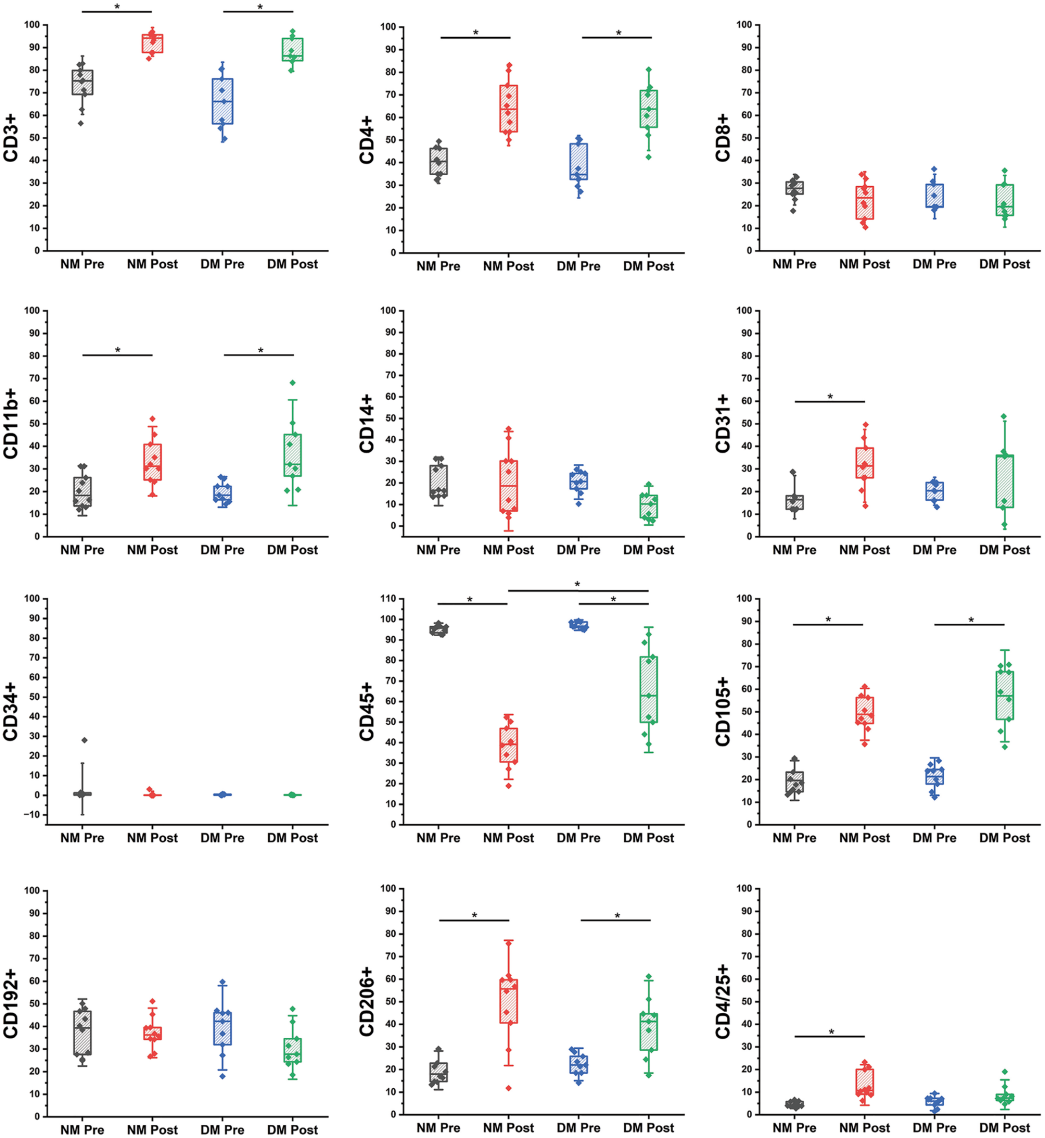
Box plots of the results of the flow cytometric analysis illustrating the proportions of cells expressing angiogenic and anti-inflammatory phenotypes, including CD3^+^, CD4^+^, CD8^+^, CD11b^+^, CD14^+^, CD31^+^, CD34^+^, CD45^+^, CD105^+^, CD192^+^, CD206^+^, and CD4^+^/25^+^. Statistical significance was assessed through ANOVA followed by Tukey's post-hoc test. In the plots, the presence of (*) denotes statistically significant differences, signifying a significance level of *p* < 0.05.

Finally, immunophenotypes were analyzed with flow cytometry to identify cell-surface markers associated with anti-inflammatory and angiogenic cell populations in the PBMCs from healthy donors and patients with DM following treatment with IronQ. The flow cytometric analysis of the scatter profile revealed a new population of cells with an increase in the side-scatter areas of PBMCs after the treatment with IronQ. Notably, the magnitude and appearance of the scatter profile did not differ between the healthy and DM groups in either the pre-IronQ or post-IronQ conditions (Supplementary Fig. S6). After the IronQ treatment, both healthy PBMCs and DM PBMCs displayed a heightened angiogenic and anti-inflammatory phenotype. We observed a substantial rise in the population of circulating endothelial progenitor cells (marked by CD11b^+^, CD31^+^, and CD105^+^), M2 macrophage cells (CD206^+^), and regulatory T cells (Tregs). In contrast, there was a decrease in the count of M1 macrophages and CD8 cells after the IronQ treatment. However, it is important to note that due to significant donor-to-donor variations, these differences did not reach statistical significance (Fig. 7).

## Discussion

In this study, we investigated the impact of IronQ, a novel MRI contrast agent, on the gene transcription profiles of PBMCs obtained from healthy donors and individuals with diabetes. Our findings revealed significant alterations in gene expression patterns induced by IronQ, which shed light on the critical pathways and genes involved in the therapeutic potential of PBMCs. The IronQ treatment led to the upregulation of genes associated with crucial regulatory mechanisms governing cell proliferation, migration, and tissue repair. We also conducted a comparative analysis to discern the similarities and differences in IronQ's effects on PBMCs from healthy and diabetic donors. These insights suggest that preconditioning PBMCs with IronQ, which is intended for tracking purposes, and augmenting their reparative capabilities could be a viable strategy. Such preconditioning may enhance the therapeutic efficacy of cell therapy by intervening in the biological processes of cultured PBMCs and optimizing their potential for reparative treatment.

Our research team previously demonstrated the effectiveness and safety of labeling PBMCs with IronQ, highlighting its potential therapeutic benefits. PBMCs can be effectively labeled with IronQ at a low concentration of 125 µg/mL, at which normal cell functions are maintained, allowing for extended cell culturing (up to 21 days). IronQ labeling of PBMCs at this concentration has been tested in vitro, and the successful application of IronQ labeling with detection facilitated through MRI has been confirmed^23^. Notably, internalizing IronQ in PBMCs at 125 µg/mL triggers their differentiation into spindle-shaped cells that express vital proangiogenic markers and augment the secretion of therapeutic factors, including angiogenic and regenerative tissue factors^17^. Integrating IronQ labeling and MRI represents a robust approach with substantial diagnostic and therapeutic implications^24^. In this study, to explore the cellular response to IronQ, we examined alterations in gene expression in PBMCs after 10 days of exposure to IronQ. The 10-day time point was deliberate, with the aim of comprehensively capturing cellular processes, including endocytic incorporation, proliferation, and early differentiation.

IronQ treatment was shown to slightly increase the production of reactive oxygen species (ROS)^18^, thus inducing oxidative stress and cellular activation. Our investigation demonstrated the upregulation of various stress-responsive and detoxifying genes, including *MGST1, HPGDS, NQO1, PRDX1, TXN, TXNRD1,* and *EPX*. These genes act as radical scavengers that protect cells from radical-induced damage. The heightened expression of these antioxidant genes indicates a cellular response to 'stress' as the cells strive to mitigate the cytotoxic effects induced by iron-induced stress^25^. Specifically, microsomal glutathione S-transferase 1 (*MGST1*) upregulation offers protection against lipid peroxidation resulting from pro-oxidant stimuli^26^. Furthermore, the increase in ROS production following IronQ treatment suggests the successful uptake of IronQ by the cells. The internalization of IronQ in cells may occur through receptor-mediated endocytosis or clathrin-dependent endocytosis, where IronQ enters cells via endocytosis and is subsequently transported to degradative machinery of the lysosome. As we observed, the generation of free radicals induced cellular stress, which likely resulted from the degradation of Fe^3+^ in IronQ due to the acidic environment of lysosomes and its subsequent release into the cytosol. In this study, we observed the upregulation of genes involved in clathrin-mediated endocytosis, including *SCARB2, CLTA, AP2A2, AP1S1, AP2M1, FCGR1A, STEAP3,* and *SNX*. These findings suggest that the uptake of IronQ in PBMCs may occur through the receptor-mediated endocytosis pathway.

Interactions between cells and nanoparticles are known to trigger a range of intracellular signaling events, including proliferation and differentiation^27,28^. In this study, we identified several enriched pathways that were common to PBMCs from healthy and diabetic donors under IronQ treatment conditions. This similarity suggests that PBMCs from these distinct sources respond similarly to IronQ, and these pathways likely play pivotal roles in IronQ induction and, resulting in the modulation of the biological processes of PBMCs. Our observations revealed notable enhancements in two key pathways—the mitogen-activated protein kinase (MAPK)/the extracellular-signal-regulated kinase (ERK) and phosphatidylinositol 3-kinase (PI3K)/protein kinase B (Akt) pathways—following IronQ treatment, which was evident through the increased expression of hub genes (*IGF1, APOE, SCARB, PPARG*, and *IL18*) associated with these pathways. These findings demonstrated the pivotal role of the stress-activated MAPK/ERK and PI3K/Akt signaling pathways in the response of PBMCs to IronQ, which was in agreement with previous research findings^29,30^.

After the IronQ treatment of PBMCs, a noteworthy log2 fold change (NM-post; log2FC = 3.67: DM-post; log2FC = 3.16) was observed in the expression of insulin-like growth factor 1 (*IGF-1*), a potent mitogenic agent that is known to bind to the IGF-1 receptor on targeted tissue cell surfaces. This binding initiated downstream signaling pathways—notably, the PI3K/Akt and the MAPK pathway. Activation of these signaling pathways is vital for various bioactivities of IGF-1, including cell proliferation, differentiation, and survival^31^. In addition to its mitogenic effects, IGF-1 also functions as an angiogenic factor. Studies have demonstrated that IGF-1 stimulates the migration of endothelial cells and promotes angiogenesis in vascular endothelial cells^32^. Furthermore, IGF-1 plays a crucial role in promoting retinal angiogenesis, with an IGF-1 receptor antagonist suppressing retinal neovascularization by inhibiting vascular endothelial growth factor (VEGF) signaling^33^. Elevated monocyte expression of IGF-1 in ischemic tissue could contribute to the angiogenic process^34^. Additionally, it has been reported that IGF-1-stimulated VEGF production is mediated by the ERK pathway in multiple myeloma cells^35^. VEGFs are central regulators of vasculogenesis and angiogenesis. VEGF-D, a secreted glycoprotein, activates VEGFR-2/VEGFR-3, exhibiting a higher affinity for VEGFR-2 and, thereby, promoting the proliferation of lymphatic endothelial cells^36^. While VEGF-A has been extensively investigated as an angiogenic factor, recent studies have shown that VEGF-D exhibits comparable angiogenic properties to those of VEGF-A in glioblastoma and colorectal cancer. VEGF-D, which is equivalent to VEGF-A, elicits the proliferation of vascular endothelial cells, thus facilitating the expansion of blood vessels that express VEGFR-2. The VEGF-D/VEGFR-2 reaction is strong, but it takes time^37^. In our previous study, we characterized circulating pro-angiogenic cells derived from PBMCs treated with IronQ and revealed the overexpression of VEGFR-2 in these treated cells^17^. Consequently, in this study, the substantial increase in *VEGF-D* expression observed in both healthy and diabetic PBMCs after IronQ treatment aligned with our prior findings, thus highlighting the role of VEGFR-2 in this angiogenic response. Matrix metalloproteinases (MMPs) are proteolytic enzymes that play a crucial role in remodeling basement membranes and the extracellular matrix (ECM), thus facilitating endothelial cell migration and invasion into the surrounding tissue during angiogenesis. Previous studies reported that IGF-1 stimulated the upregulation of MMPs^38,39^, which aligned with our findings concerning the significant increase in *MMP2* and *MMP9* expression in PBMCs treated with IronQ. Therefore, we postulate that when IGF-1 stimulates the secretion of pro-angiogenic factors by PBMCs, it initiates endothelial cell growth, migration, and vascularization. Recent evidence indicated that dysfunction in endothelial progenitor cells (EPCs) in diabetic patients adversely affected vasculogenesis and hampered efficient wound regeneration^22^. Our study demonstrated that pre-treatment with IronQ can rejuvenate the pro-angiogenic functionality of PBMCs in diabetic patients.

The preconditioning of PBMCs—whether they were derived from healthy or diabetic donors—with IronQ significantly enriched their immune regulatory response. Research has shown that exposure to cellular-stress-inducing stimuli activates repair mechanisms and immune regulatory responses in mesenchymal stem cells (MSCs) and macrophages^40,41^. In our study, treating PBMCs with IronQ led to a notable increase in the expression of colony-stimulating factor 1 (*CSF-1*), another critical hub gene identified in our analyses. CSF-1, also known as macrophage (M)-CSF, plays a central role in regulating macrophage differentiation, proliferation, and survival. It acts as a hemopoietic growth factor for the mononuclear phagocyte lineage and holds therapeutic potential for tissue repair. In the realm of tissue repair, CSF-1 presents therapeutic potential. Recent work by Alikhan et al. highlighted the ability of CSF-1 to promote renal repair following reversible ischemia. Their study demonstrated that CSF-1 treatment augmented macrophage recruitment, polarized cells toward an M2-like phenotype, and encouraged the production of growth factors, including IGF-1^42^.

Moreover, monocytes treated with CSF-1 exhibited potential transdifferentiation into endothelium in vitro. In contrast, CSF-1 primarily promoted the production of pro-angiogenic growth factors in vivo^43^. It is well known that M2 macrophages have been attributed functions in immunosuppression and vascularization in tumor environments^44^. The regulation of the macrophage phenotype is crucial for coordinating tissue injury recovery and efficient wound healing in various tissues^45–47^. The IronQ treatment of PBMCs stimulated the polarization of M2-like macrophages, which was evident in the increased expression of M2 macrophage phenotypes (*CD206*, *CD163*, and *CD209*) and the upregulation of genes associated with anti-inflammatory cytokines and chemokines, including *IL10*, *CCL-17*, and *IDO,* after the treatment. Additionally, we noted the upregulation of crucial transcription factors such as *PPARG* (PPARγ), which regulates the expression of M2 macrophage genes^48^. These results align with our previous findings, where the IronQ preconditioning of MSCs exhibited its anti-inflammatory effects by inhibiting the activity of the Mincle/syk signaling pathway, suppressing inflammatory factor production in macrophages, and promoting their polarization into M2 macrophages in mouse models of intracerebral hemorrhage (ICH)^24^.

In addition to promoting angiogenesis, the IGF-1 signaling pathway significantly influences reparative functions by regulating the innate and adaptive immune systems. Evidence suggests that IGF-1 signaling orchestrates anti-inflammatory and immunosuppressive responses^49^. Signal transducer and activator of transcription 3 (STAT3), a central transcription factor in this context, plays a pivotal role in stimulating immunosuppressive cells, including myeloid-derived suppressor cells (MDSCs) and regulatory T cells (Tregs), particularly under conditions of chronic inflammation^50,51^. The key signaling pathways stimulated by IGF-1 that govern immunosuppression involve the activation of STAT3 via Akt kinase and the mammalian target of rapamycin (mTOR)^52^. In our study, a significant increase in *FOXP3* expression was observed in PBMCs under IronQ treatment conditions. FOXP3 is a crucial transcription factor involved in the genetic programming of CD4^+^CD25^+^ Treg differentiation and function^53^. Previous studies confirmed that STAT3 activation directly acts on CD4^+^ T lymphocytes to promote FOXP3 expression. These findings align with the observation of decreased Tregs following STAT3 ablation in hemopoietic cells^54^. Based on this, we hypothesize that the IronQ treatment of PBMCs stimulates an increase in Tregs by activating IGF-1 signaling and, thus, engaging in crosstalk with STAT3 signaling activation in T lymphocytes to promote the expression of Treg cells. Furthermore, we observed an upregulation of *CD274*, which is commonly referred to as PD-L1 (programmed death-ligand 1), following the IronQ treatment of PBMCs. Previous studies demonstrated that PD-L1 and PD-L2 play a vital role in immune regulation by suppressing T cell activation and promoting the induction of Tregs^55^. It is known that, under pro-inflammatory stimulation, MSCs can increase the expression of PD-1 ligands, leading to enhanced suppression of T cell effector function^56,57^. In this study, we demonstrated that IronQ treatment significantly increased PD-L1 expression in PBMCs, resulting in an enhanced Treg population, as validated through cell phenotype analysis with immunocytometric techniques. These findings emphasize the crucial role of PD-1 ligands in mediating Treg induction.

It is noteworthy that treatment with IronQ significantly increased the expression of interleukin18 (*IL18*)—also known as interferon-gamma inducing factor—in PBMCs. IL18 is a versatile cytokine secreted by dendritic cells, macrophages, and epithelial cells, and it is crucial for interferon production by T cells and natural killer cells (NK)^58^. IL18 is unique, as it exhibits a duality in its effects. On the one hand, in conjunction with IL12, it stimulates robust host immune responses, and it mainly promotes Th1 responses, which are characterized by the activation and proliferation of cytotoxic T cells. Conversely, at lower doses and in the absence of IL12, IL18 exerts pro-cancer effects by inducing immune evasion and promoting angiogenesis^59^. Recent findings indicated that IL18 treatment significantly augmented the proportion and absolute count of monocytic myeloid-derived suppressor cells (M-MDSCs) by differentiating CD11b^-^ progenitor cells. This elevation in IL18-induced MDSCs led to the suppression of T cell proliferation and IFN production, thus contributing to the immunosuppressive properties associated with IL18^60^. The effective coordination of various immune cells that govern local immunomodulation can reshape the molecular environment and amplify tissue repair processes^61^. These findings suggest that enhancing the immunomodulatory potential of PBMCs by preconditioning them with IronQ may significantly augment the therapeutic efficacy of PBMCs in the reparative context.

A notable observation in this study was that under IronQ treatment conditions, PBMCs from both healthy and diabetic donors exhibited a reduction in the expression of pro-inflammatory genes, such as *CD8A*, *CD69*, *GZMB*, *CCL2*, *CX3CR1*, and *CCR5*. These genes play critical roles in biological processes associated with the inflammatory response, including T cell proliferation and activation, leukocyte differentiation, and natural-killer-cell-mediated cytotoxicity. The downregulation of these genes suggests a decrease in inflammatory activity. As described in the findings above, this potential reduction in the pro-inflammatory response may be linked to the immunosuppressive properties induced by IronQ treatment in PBMCs. This impact of IronQ on the immunomodulatory function of PBMCs highlights their potential application in repairing tissues that are characterized by chronic inflammation.

It is intriguing to observe that the IronQ treatment affected different pathways in PBMCs from different sources. Notably, in PBMCs derived from diabetic donors, the IronQ treatment significantly enriched pathways related to the cell cycle, including those of cell division, cell proliferation, and cellular senescence, as evidenced by the increased expression of cell-cycle-related genes such as *CCND1*, *PKMYT1*, *CDC20*, *BUB1B*, *CCNB2*, *BUB1*, *CDC45*, *CDK1*, *CDC25*, and *PTTG*. Moreover, the p53 signaling pathway, which is associated with cell senescence, was substantially enriched, with increased transcription of p53-related genes such as *RRM2* and *CDK1*. This distinction may be attributed to the persistent hyperglycemia seen in diabetic patients, which impacts the regulation of cell proliferation and metabolic processes. The protein O-linked N-acetylglucosamine (O-GlcNAc) modification, which is considered to play a significant role in the deleterious effects of hyperglycemia, is implicated in this phenomenon^62^. Numerous cell cycle regulators, including cyclin D1, cyclin-dependent kinase 1 (CDK1), cyclin-dependent kinase 4 (CDK4), and cell division cycle-25 (CDC25), have been identified as O-GlcNAc substrates, and their functions are affected by O-GlcNAc levels^63^. Sustained hyperglycemia increases glycolytic efflux, leading to elevated O-GlcNAc production and, subsequently, causing abnormal cell proliferation in PBMCs from diabetic donors. Our findings align with those of previous research, which showed the upregulation of cell cycle genes in various types of cells from diabetic patients, including neurons, endothelial cells, and blood cells^64–66^. Importantly, our study indicated that IronQ did not influence the metabolic modification processes of PBMCs from diabetic donors.

Another notable difference observed under IronQ treatment conditions was the significant enrichment of the IL17 signaling pathway among the DEGs unique to healthy PBMCs, with considerable upregulation of core genes in this pathway such as *CXCL3*, *CCL2*, *MMP9*, *CXCL1*, *CCL7*, *MAPK13*, *TRAF2*, *TRAF3*, *IL17RE*, *CXCL2*, and *CHUK* following the IronQ treatment. These changes were not observed in diabetic PBMCs. The activation of the IL17 signaling pathway was able to stimulate the downstream nuclear factor-кB (NF-кB) and MAPK signaling pathways, resulting in the enhanced expression of antimicrobial peptides, cytokines, chemokines, and tissue remodeling compounds. This pathway plays a pivotal role in recruiting additional proinflammatory immune cells, promoting the proliferation of keratinocytes and, ultimately, aiding in acute wound healing. IL17 signaling has been shown in numerous studies to induce the release of proinflammatory cytokines such as tumor necrosis factor-alpha (TNF-α), IL6, and IL1, as well as TNF-α signaling, thus mediating the production of inflammatory molecules that are crucial for epithelial wound healing. TNF-α/TNF-R2 signaling has been demonstrated to enhance epithelial migration and boost the survival and proliferation of intestinal epithelial cells (IECs)^67^. Furthermore, TNF-α promotes re-epithelialization and wound healing by upregulating the production of FGF-7^68^. Our validation results substantiate these findings, showing increased mRNA expression of *CXCL3*, *CCL2*, and *TNF-α* in PBMCs treated with IronQ. Improved local cell survival is typically associated with enhanced repair and recovery^69^. Interestingly, the term “pathways in cancer” was significantly enriched with upregulated genes after the IronQ treatment of PBMCs. Given the overlap in molecular mechanisms between cancer and regenerative biology, particularly in pathways such as mTOR and Wnt signaling, genes involved in these overlapping pathways—such as *PDGFB*, *TGFA*, *NRP1*, *EPHB2*, *WNT5A*, *RRAS*, *NCK1*, *WNT5B*, *PLXNA1*, *POU5F1*, and *LRP5*—could potentially contribute to tissue repair. Our findings align with those of previous research that indicated the role of Wnt signaling in stem cell recruitment and differentiation during wound repair, suggesting that modulating Wnt signaling positively impacts tissue repair^70,71^. Our data indicated that the IronQ treatment of healthy PBMCs triggered more robust paracrine survival mechanisms than those in untreated healthy PBMCs. Thus, enhancing the ability of PBMCs to support cell survival with IronQ before transplantation could lead to improved transplant-mediated tissue repair.

Finally, we validated the expression levels of anti-inflammatory, pro-angiogenic, and wound-healing-associated genes to substantiate our findings from the RNA-seq analysis. Additionally, we characterized the cell phenotype of PBMCs after IronQ treatment using specific surface markers associated with anti-inflammatory and pro-angiogenic properties. As expected, our results indicated that PBMCs from diabetic patients exhibited a pro‐inflammatory state. At baseline, the numbers of cells that were positive for T cell and Treg markers did not differ significantly between healthy and diabetic PBMCs. However, diabetic PBMCs expressed higher levels of pro-inflammatory genes such as leukemia-inhibitory factor (LIF) and TNF-α. Intriguingly, we also observed lower levels of angiogenic T cell markers in diabetic PBMCs, suggesting that PBMCs from diabetic patients presented a highly inflammatory and antiangiogenic phenotype compared to those from healthy individuals. After the IronQ treatment, both healthy and diabetic PBMCs demonstrated an increase in angiogenic T cells, pro-angiogenic progenitors, M2 macrophages, and Treg cells. Conversely, there was a significant decrease in cytotoxic T cells (CD8^+^) after the IronQ treatment. This observation aligned with our qRT-PCR results, where we observed the upregulation of anti-inflammatory cytokines and genes associated with pro-angiogenesis and wound healing. The products of these genes likely act as positive regulators, accelerating vasculogenesis and tissue repair processes. Interestingly, we observed that diabetic PBMCs exhibited elevated levels of LIF following IronQ treatment. LIF, a cytokine in the IL6 superfamily, plays a crucial role in regulating cell division, proliferation, and survival^72^. Recent studies have highlighted the potential of upregulated LIF expression as a promising approach to enhancing the proangiogenic capabilities of MSCs^73^. In addition, the expression of autophagy-associated genes was noticeably upregulated in healthy and diabetic PBMCs after the IronQ treatment, with a more pronounced increase being observed in healthy PBMCs. Recent research has underscored the critical role of autophagy in enhancing the survival of transplanted stem cells and augmenting the therapeutic potential of stem-cell-based therapies^74,75^. In a recent study involving a rat model of ischemia–reperfusion myocardial injury, incubating bone-marrow-derived MSCs with rapamycin activated their autophagic processes, affording protection against apoptosis and improving their survival and differentiation after transplantation into ischemic myocardial areas^76^. Hence, pre-treating PBMCs with IronQ may activate the autophagic process, thus bolstering their survival in challenging microenvironments and amplifying their therapeutic potential in cell-based therapies.

Notably, there were no notable differences in the prospective angiogenic and tissue repair properties of IronQ-treated PBMCs from healthy donors and diabetic patients. These findings strengthen the rationale for considering the pretreatment of PBMCs from diabetic patients as a strategy for augmenting treatment efficacy, thus further emphasizing the promising potential of autologous cell therapy. Nevertheless, our study has certain limitations that warrant consideration in future research. These limitations include the small sample sizes, the necessity of assessing the therapeutic efficacy of PBMCs preconditioned with IronQ in both in vitro and in vivo settings, and the importance of involving more diverse and representative populations in subsequent studies to comprehensively grasp the potential applications of preconditioning with IronQ and PBMCs.

## Materials and methods

### Preparation and characterization of IronQ

Dr. Nathupakorn Dechsupa from the Molecular Imaging and Therapy Research Unit, Department of Radiologic Technology, Faculty of Associated Medical Sciences, Chiang Mai University generously provided the iron (III)–quercetin complex (IronQ). IronQ has a hydrodynamic dimension of 160.0 ± 2.4 nm in Milli-Q water and is known for its spherical morphology. Notably, its surface carries a negative charge, as evidenced by its mean zeta potential of − 24.53 ± 1.88 mV, indicating its moderate stability when suspended in water. This negative zeta potential reflects the ionic charge in IronQ, which generally extends the complex's half-life in the bloodstream by facilitating electrostatic repulsion with plasma proteins. IronQ was dissolved in distilled water to produce an IronQ stock solution with an intended ultimate concentration of 2 mg/mL. Subsequently, the IronQ solution underwent filter sterilization with a sterile 0.2 µm syringe filter before use.

### Human subject

A total of ten male patients, aged between 30 and 70 years, diagnosed with type 2 diabetes and having HbA1C levels below 8.0 g/dL, were recruited from the private healthcare clinic in the local area of Chiang Mai province, Thailand. Exclusion criteria encompassed patients with malignant tumors, those undergoing hemodialysis or peritoneal dialysis, and individuals suffering from infectious diseases, hematologic disorders, or severe heart failure. Additionally, we selected ten healthy male volunteers, aged between 30 and 70 years, as control subjects. This study's design received approval from the Human Research Ethics Committee of the Faculty of Associated Medical Sciences at Chiang Mai University (protocol no. AMSEC-65EX-023, dated 7 July 2022). All experiments were conducted following the guidelines and regulations approved by the Review Board of this committee and adhered to the principles of the Declaration of Helsinki. Before they participated in this research, all participants provided informed consent.

### Isolation and cell culture of peripheral blood mononuclear cells (PBMCs)

Healthy individuals and patients diagnosed with diabetes mellitus (DM) contributed 50 mL of whole blood samples; then, the density gradient centrifugation method was employed to successfully isolate the peripheral blood mononuclear cells (PBMCs). Specifically, phosphate buffer saline (PBS, pH 7.4) was used to dilute the blood samples in an equivalent proportion. Subsequently, the diluted blood was carefully layered on top of lymphocyte separation media (Lymphoprep™, Stem Cell Technologies, Vancouver, BC, Canada), and the mixture was then centrifuged. This process resulted in the formation of a distinct white layer consisting of mononuclear cells. These mononucleated cells were gathered and cultured in Roswell Park Memorial Institute 1640 (RPMI 1640) medium supplemented with L-glutamine (Caisson Lab, Smithfield, UT, USA), 10% fetal bovine serum (FBS) (Gibco, Waltham, MA, USA), and 1% penicillin/streptomycin (Gibco, Waltham, MA, USA). Afterwards, the cells were cultivated in an incubator with 5% CO_2_ in a humid atmosphere.

### Treatment of PBMCs with IronQ and observation of their morphology

PBMCs that were freshly isolated from healthy and DM patients were cultured in cell culture flasks at 1 × 10^6^ cells/mL in RPMI 1640 culture media supplemented with 10% FBS. The cells were then exposed to IronQ at a concentration of 125 µg/mL. The selection of the IronQ concentration of 125 µg/mL for the treatment was based on a previous validation demonstrating the practical potential of PBMCs at this dosage^17^. Culturing was conducted at 37 °C in a humidified atmosphere with 5% CO_2_ and without any subculture or re-feeding. After 10 days of incubation, the post-IronQ treatment cells were collected via careful pipetting, and the wells were rinsed with EDTA-PBS solution. Alterations in cell morphology were observed before collection on day 10 by using an inverted microscope. Subsequently, the collected cells were suspended in an appropriate medium for further experiments.

### RNA-sequencing analysis

After collection, the freshly isolated PBMCs and those treated with IronQ for ten days were processed as follows. The isolation of total RNA was performed by using the Nucleospin® RNA Kit (Macherey–Nagel, Düren, Germany) according to the manufacturer's instructions. This extracted RNA was treated with rDNase—a product that was RNase-free—to eliminate any genomic DNA contamination. The RNA quantity was ascertained by utilizing a Nanodrop spectrophotometer (BioDrop™ μLITE, Cambridge, UK), while the integrity of the RNA was evaluated through the computation of the A260/A280 ratio. Messenger RNA (mRNA) was purified from total RNA via oligo dT selection and fragmentation. It was then reverse-transcribed into first-strand cDNA by using random hexamer primers and synthesized into double-stranded cDNA, with the first-strand cDNA as the template. After the end repair process, the double-stranded cDNA underwent polymerase chain reaction (PCR), amplification, and subsequent purification to create a cDNA library. Quantified libraries were combined and subjected to RNA sequencing on Illumina platforms, with a focus on optimal library concentration and data volume. The resulting reads had a length of 150 base pairs. The raw sequencing data were processed to generate clean reads by eliminating low-quality reads and reads containing ploy-N or adaptor sequences by using FastQC^77^. The clean data were then assessed for quality by calculating the Q20, Q30, and GC content. Subsequently, the high-quality filtered data were aligned with the human reference genome (GRCh38) by using Hisat2 (v2.0.5).

Gene expression was measured by using featureCounts v1.5.0-p3, and the results were displayed as FPKM or fragments per kilobase of transcript sequence per million mapped reads. A principal component analysis (PCA) algorithm was applied to evaluate variations in mRNA expression among the different groups. Differentially expressed genes (DEGs) were identified by using the DESeq2 R package (version 1.20.0), which assessed the data based on the negative binomial distribution. Significant DEGs were defined as those with a Q value (adjusted p-value calculated by using the Benjamini and Hochberg method) of less than 0.05 and an absolute log2-fold change (FC) greater than or equal to 1. Volcano plots and expression heatmaps were generated by using web-based analytical tools (NovoMagic, a free Novogene platform).

### Bioinformatic analysis of the differentially expressed genes (DEGs)

We comprehensively analyzed the DEGs obtained from the various comparisons in our study. We employed a Venn diagram tool to identify common DEGs across different comparisons. The functional enrichment analysis of these DEGs was carried out by using the NovoMagic, a free Novogene platform for data analysis and the assessment of Gene Ontology (GO) terms and KEGG pathways^78^(http://www.genome.jp/kegg/). Significance was determined based on Q values (adjusted *p*-value) that were less than 0.05. Furthermore, we constructed a protein–protein interaction (PPI) network by using the STRING database (https://www.string-db.org/) and visualized it with Cytoscape version 3.8.2 (https://cytoscape.org/). We utilized the Molecular Complex Detection (MCODE) and CytoHubba plug-ins in Cytoscape to identify key clusters and hub genes in the PPI network. The functions of genes in these clusters were further analyzed by using the Metascape database (http://metascape.org). The results of these analyses were represented in heatmaps that highlighted enriched terms and provided valuable insights into the functional roles and interactions of the identified genes.

### Quantitative real-time polymerase chain reaction (qRT-PCR)

Peripheral blood mononuclear cells (PBMCs) that were freshly isolated and those that were subjected to a ten-day IronQ treatment were processed as follows. Total RNA isolation was performed ’by using the Nucleospin® RNA Kit (Macherey–Nagel, Düren, Germany) according to the manufacturer’s protocol. The isolated RNA was utilized to synthesize complementary DNA (cDNA) with the ReverTra Ace™ qPCR RT Master Mix, including a gDNA Remover (TOYOBO, Osaka, Japan), per the manufacturer's instructions. Quantitative real-time polymerase chain reaction (qRT-PCR) was carried out on a CFX ConnectTM real-time system (Bio-Rad, California, USA) by using cDNA mixed with THUNDERBIRDTM Next SYBR® qPCR Mix (TOYOBO, Osaka, Japan), with the following cycling conditions: an initial denaturation at 95 °C for 60 s, followed by denaturation at 95 °C for 10 s, annealing at 61.1, 62.4, 64.9, and 66.1 °C for 15 s, and extension at 72 °C for 30 s. This cycle was repeated 40 times, and the reactions were performed in triplicate. The primer sequences used in this study are presented in Table 1. Normalization of the calculated Ct values of the target genes was performed by using the housekeeping gene GAPDH. The 2^-∆∆Ct^ technique was utilized to assess the relative gene expression in order to quantify the fold changes in gene expression between the treatment and control groups. In total, 7 healthy donors and 7 diabetic donors were examined independently from RNA-Sequencing Analysis samples.

**Table 1:** List of primers used in quantitative real-time polymerase chain reaction (qRT-PCR).

Target	Forward primer (5′–3′)	Reverse primer (5′–3′)
*CCL17*	GAGCCATTCCCCTTAGAAAGC	CTCTCTTGTTGTTGGGGTCC
*CCL2*	CCAGTCACCTGCTGTTATAAC	GTGAGTGTTCAAGTCTTCGG
*CXCL3*	CTAGGGACAGCTGGAAAGGAC	CTGACACATTATGGTCTCCCAC
*IDO*	CAGCTATCAGACGGTCTGGT	CAGGAAGTTCCTGTGAGCTG
*LC3B*	GCCTGAGTTGTGAAGCGCAA	CTTAGGAGTCAGGGACCTTC
*IL10*	CTGAGCTTCTCTGTGAACGA	CAGCTAGAAAGCGTGGTCAG
*TNFA*	GTACCTCATCTACTCCCAGG	CAGATAGATGGGCTCATACC
*TGFB*	GGTACCTGAACCCGTGTTGC	CGCACAACTCCGGTGACATC
*MMP2*	GACGGAAAGATGTGGTGTGC	CCCCATAGAGCTCCTGAATG
*mTOR*	CCTGTCAGAATCCAAGTCAAG	GGGACTTGAAGATGAAGGTG
*AKT*	CGGAGAAGAACGTGGTGTACC	GGCCGTAGTCATTGTCCTCC
*ATG7*	CTTCTGCAATGATGTGGTGGC	CGTCACTGCTGCTGGCAATG
*Beclin1*	GAGGCTGAGAGACTGGATCAG	GTGGAAGGTTGCATTAAAGACG
*Bnip3l*	GGATGACCAGTTATCTCGAG	GGCAAACCTGTCTGTCATAG
*LIF*	GCCCCCAGCAAATTATCACC	CCTCCCCAAGAGCCTGAATG
*GAPDH*	GTATCGTGGGAAGGACTCATGAC	GAACATCATCCCTGCCTCTAC

### Analysis of cell surface markers using flow cytometry

Freshly isolated PBMCs and those treated with IronQ were collected in PBS and subsequently blocked for 30 min at 4 °C by using a blocking reagent (PBS containing 2.5% BSA). Cell suspensions were subjected to staining in PBS containing 0.1% BSA at a concentration of 5 × 10^5^ cells. Each 100 µL sample of the cell suspension was incubated with the appropriate IgG isotype controls for each fluorescence channel or with specific antibodies, including FITC-labeled anti-CD34 (Elabscience), FITC-labeled anti-CD14 (Miltenyi), FITC-labeled anti-CD11b (eBioscience), PE/Cy5.5‐labeled anti-CD45 (eBioscience), FITC-labeled anti-CD31 (Life Technologies), PE-labeled anti-CD105 (eBioscience), PE/Cy5.5‐labeled anti‐CD3(clone: UCHT1, Merck Millipore), APC‐labeled anti‐CD206 (clone 15–2, Sigma-Aldrich), APC‐labeled anti‐CCR2 (CD192, Sino Biological), PE‐labeled anti‐CD8a (clone: RPA‐T8, Merck Millipore), PE‐labeled anti‐CD4 (clone: RPA‐T4, Merck Millipore), and Alexa Fluor‐488‐labeled anti‐CD25 (Clone: BC96, eBioscience). This incubation was performed at 4 °C in the dark for 30 min. After two washes containing 0.1% BSA with PBS, the cell suspension was subjected to analysis by using flow cytometry (Beckman Coulter, Epics XL-MCL, CA, USA). Data analysis was carried out by using the FlowJo10 software (Tree Star, Ashland, Oregon), and scattergrams were gated based on three different cell sizes.

### Statistical analysis

The data from qRT-PCR and flow cytometry are presented as mean values accompanied by the standard deviation (SD). Each study included ten individual donors per group. The analysis of statistical differences among the treatment groups was assessed through one-way ANOVA, followed by Tukey's multiple-comparison post-hoc tests with OriginPro version 2023 (Origin Lab, Northampton, MA, USA). It was determined that a *p*-value below 0.05 indicated significance.

### Supplementary Information

Supplementary Figures.

Supplementary Table 1.

Supplementary Table 2.

Supplementary Table 3.

Supplementary Table 4.

## Data Availability

The datasets analyzed during the present study have been deposited in the National Center for Biotechnology Information Sequence Read Archive (NCBI SRA) under accession code PRJNA1090784. Additional data generated or analyzed during this study are included in its supplementary information files or available from the corresponding author on reasonable request.

## References

[1] Eming, S. A., Martin, P. & Tomic-Canic, M. Wound repair and regeneration: mechanisms, signaling, and translation. *Sci. Transl. Med*. **6,** 265sr266 (2014). 10.1126/scitranslmed.3009337 PMC4973620 25473038

[2] El-Kadiry, A. E., Rafei, M. & Shammaa, R. Cell therapy: Types, regulation, and clinical benefits. *Front Med (Lausanne).* **8**, 756029 (2021). 10.3389/fmed.2021.756029 PMC8645794 34881261

[3] Hoang DM (2022). Stem cell-based therapy for human diseases. Signal Transduct Target Ther..

[4] Zhuang W-Z (2021). Mesenchymal stem/stromal cell-based therapy: Mechanism, systemic safety and biodistribution for precision clinical applications. J. Biomed. Sci..

[5] Chong PP, Selvaratnam L, Abbas AA, Kamarul T (2012). Human peripheral blood derived mesenchymal stem cells demonstrate similar characteristics and chondrogenic differentiation potential to bone marrow derived mesenchymal stem cells. J. Orthop. Res..

[6] Zhu Y, Fu W (2022). Peripheral blood-derived stem cells for the treatment of cartilage injuries: A systematic review. Front. Bioeng. Biotechnol..

[7] Chuang CH (2023). Enriched peripheral blood-derived mononuclear cells for treating knee osteoarthritis. Cell Transplant..

[8] Hopper N (2015). Peripheral blood mononuclear cells enhance cartilage repair in in vivo osteochondral defect model. PLOS ONE..

[9] Blocki A (2015). Sourcing of an alternative pericyte-like cell type from peripheral blood in clinically relevant numbers for therapeutic angiogenic applications. Mol. Ther..

[10] Li S (2015). Peripheral blood-derived mesenchymal stem cells: candidate cells responsible for healing critical-sized calvarial bone defects. Stem Cells Transl Med..

[11] Baraniak PR, McDevitt TC (2010). Stem cell paracrine actions and tissue regeneration. Regen. Med..

[12] Gnecchi M, Zhang Z, Ni A, Dzau VJ (2008). Paracrine mechanisms in adult stem cell signaling and therapy. Circ. Res..

[13] Beer L (2015). Analysis of the secretome of apoptotic peripheral blood mononuclear cells: Impact of released proteins and exosomes for tissue regeneration. Sci. Rep..

[14] Mildner M (2013). Secretome of peripheral blood mononuclear cells enhances wound healing. PLOS ONE..

[15] Ankersmit HJ (2009). Irradiated cultured apoptotic peripheral blood mononuclear cells regenerate infarcted myocardium. Eur. J. Clin. Invest..

[16] Hoetzenecker K (2015). Mononuclear cell secretome protects from experimental autoimmune myocarditis. Eur. Heart J..

[17] Kantapan J (2021). Iron-quercetin complex preconditioning of human peripheral blood mononuclear cells accelerates angiogenic and fibroblast migration: Implications for wound healing. Int J Mol Sci..

[18] Dechsupa N (2022). Iron(III)–quercetin complexes’ safety for MRI cell tracking in cell therapy applications: Cytotoxic and genotoxic assessment. Nanomaterials..

[19] Patel S, Srivastava S, Singh MR, Singh D (2019). Mechanistic insight into diabetic wounds: Pathogenesis, molecular targets and treatment strategies to pace wound healing. Biomed. Pharmacother..

[20] Tanaka R (2014). Autologous G-CSF-mobilized peripheral blood CD34+ cell therapy for diabetic patients with chronic nonhealing ulcer. Cell Transplant..

[21] Shin L, Peterson DA (2012). Impaired therapeutic capacity of autologous stem cells in a model of type 2 diabetes. Stem Cells Transl. Med..

[22] Fadini GP (2013). Diabetes impairs stem cell and proangiogenic cell mobilization in humans. Diabetes Care.

[23] Papan P, Kantapan J, Sangthong P, Meepowpan P, Dechsupa N (2020). Iron (III)-quercetin complex: Synthesis, physicochemical characterization, and MRI cell tracking toward potential applications in regenerative medicine. Contrast Media Mol. Imaging..

[24] Yang G (2023). Mesenchymal stem cells transplantation combined with IronQ attenuates ICH-induced inflammation response via Mincle/syk signaling pathway. Stem Cell Res. Ther..

[25] Tavleeva, M. M. *et al.* Effects of antioxidant gene overexpression on stress resistance and malignization in vitro and in vivo: A Review. *Antioxidants (Basel).* **11,** (2022). 10.3390/antiox11122316 PMC9774954 36552527

[26] Peng Z, Peng N (2023). Microsomal glutathione S-transferase 1 targets the autophagy signaling pathway to suppress ferroptosis in gastric carcinoma cells. Hum. Exp. Toxicol..

[27] Rauch J, Kolch W, Mahmoudi M (2012). Cell type-specific activation of AKT and ERK signaling pathways by small negatively-charged magnetic nanoparticles. Sci. Rep..

[28] Wei M, Li S, Le W (2017). Nanomaterials modulate stem cell differentiation: biological interaction and underlying mechanisms. J Nanobiotechnol..

[29] Rojas JM (2016). Superparamagnetic iron oxide nanoparticle uptake alters M2 macrophage phenotype, iron metabolism, migration and invasion. Nanomedicine..

[30] Wang Q (2016). Response of MAPK pathway to iron oxide nanoparticles in vitro treatment promotes osteogenic differentiation of hBMSCs. Biomaterials..

[31] Hakuno F, Takahashi SI (2018). IGF1 receptor signaling pathways. J. Mol. Endocrinol..

[32] Nakao-Hayashi J, Ito H, Kanayasu T, Morita I, Murota S (1992). Stimulatory effects of insulin and insulin-like growth factor I on migration and tube formation by vascular endothelial cells. Atherosclerosis..

[33] Smith LE (1999). Regulation of vascular endothelial growth factor-dependent retinal neovascularization by insulin-like growth factor-1 receptor. Nat. Med..

[34] Kluge A (1997). Coordinate expression of the insulin-like growth factor system after microembolisation in porcine heart. Cardiovasc. Res..

[35] Menu E (2004). Specific roles for the PI3K and the MEK–ERK pathway in IGF-1-stimulated chemotaxis, VEGF secretion and proliferation of multiple myeloma cells: Study in the 5T33MM model. Br. J. Cancer..

[36] Orlandini M, Marconcini L, Ferruzzi R, Oliviero S (1996). Identification of a c-fos-induced gene that is related to the platelet-derived growth factor/vascular endothelial growth factor family. Proc. Natl. Acad. Sci. U S A..

[37] Jia H (2004). Vascular endothelial growth factor (VEGF)-D and VEGF-A differentially regulate KDR-mediated signaling and biological function in vascular endothelial cells. J. Biol. Chem..

[38] Mira E, Mañes S, Lacalle RA, Márquez G, Martínez AC (1999). Insulin-like growth factor I-triggered cell migration and invasion are mediated by matrix metalloproteinase-9. Endocrinology..

[39] Grzmil M, Hemmerlein B, Thelen P, Schweyer S, Burfeind P (2004). Blockade of the type I IGF receptor expression in human prostate cancer cells inhibits proliferation and invasion, up-regulates IGF binding protein-3, and suppresses MMP-2 expression. J. Pathol..

[40] Li M (2022). Potential pre-activation strategies for improving therapeutic efficacy of mesenchymal stem cells: Current status and future prospects. Stem Cell Res. Ther..

[41] Wunderlich R (2015). Low and moderate doses of ionizing radiation up to 2 Gy modulate transmigration and chemotaxis of activated macrophages, provoke an anti-inflammatory cytokine milieu, but do not impact upon viability and phagocytic function. Clin. Exp. Immunol..

[42] Alikhan MA (2011). Colony-stimulating factor-1 promotes kidney growth and repair via alteration of macrophage responses. Am. J. Pathol..

[43] Okuno Y, Nakamura-Ishizu A, Kishi K, Suda T, Kubota Y (2011). Bone marrow-derived cells serve as proangiogenic macrophages but not endothelial cells in wound healing. Blood..

[44] Boutilier, A. J. & Elsawa, S. F. Macrophage polarization states in the tumor microenvironment. *Int. J. Mol. Sci.* **22,** (2021). 10.3390/ijms22136995 PMC8268869 34209703

[45] Xie, X. *et al.* Trib1 contributes to recovery from ischemia/reperfusion-induced acute kidney injury by regulating the polarization of renal macrophages. *Front. Immunol.* **11,** (2020). 10.3389/fimmu.2020.00473 PMC7098949 32265926

[46] Wang Y (2023). Umbilical cord mesenchymal stem cell-derived apoptotic extracellular vesicles ameliorate cutaneous wound healing in type 2 diabetic mice via macrophage pyroptosis inhibition. Stem Cell Res. Ther..

[47] Tacke F, Zimmermann HW (2014). Macrophage heterogeneity in liver injury and fibrosis. J. Hepatol..

[48] Murray PJ (2017). Macrophage polarization. Annu. Rev. Physiol..

[49] Labandeira-Garcia JL, Costa-Besada MA, Labandeira CM, Villar-Cheda B, Rodríguez-Perez AI (2017). Insulin-like growth factor-1 and neuroinflammation. Front. Aging Neurosci..

[50] Ko H-J, Kim Y-J (2016). Signal transducer and activator of transcription proteins: regulators of myeloid-derived suppressor cell-mediated immunosuppression in cancer. Arch. Pharm. Res..

[51] Sun G (2017). STAT3 promotes bone fracture healing by enhancing the FOXP3 expression and the suppressive function of regulatory T cells. Apmis..

[52] Salminen A, Kaarniranta K, Kauppinen A (2021). Insulin/IGF-1 signaling promotes immunosuppression via the STAT3 pathway: Impact on the aging process and age-related diseases. Inflamm. Res..

[53] Rudensky AY (2011). Regulatory T cells and Foxp3. Immunol. Rev..

[54] Kortylewski M (2005). Inhibiting Stat3 signaling in the hematopoietic system elicits multicomponent antitumor immunity. Nat. Med..

[55] Cai J, Wang D, Zhang G, Guo X (2019). The role of PD-1/PD-L1 axis in Treg development and function: Implications for cancer immunotherapy. Onco Targets Ther..

[56] Gonçalves FDC (2017). Membrane particles generated from mesenchymal stromal cells modulate immune responses by selective targeting of pro-inflammatory monocytes. Sci. Rep..

[57] Hackel, A., Vollmer, S., Bruderek, K., Lang, S. & Brandau, S. Immunological priming of mesenchymal stromal/stem cells and their extracellular vesicles augments their therapeutic benefits in experimental graft-versus-host disease via engagement of PD-1 ligands. *Front Immunol.* **14**, 1 (2023). 10.3389/fimmu.2023.1078551 PMC9978482 36875112

[58] Ihim SA (2022). Interleukin-18 cytokine in immunity, inflammation, and autoimmunity: Biological role in induction, regulation, and treatment. Front. Immunol..

[59] Fabbi M, Carbotti G, Ferrini S (2015). Context-dependent role of IL-18 in cancer biology and counter-regulation by IL-18BP. J. Leukoc. Biol..

[60] Lim HX, Hong HJ, Cho D, Kim TS (2014). IL-18 enhances immunosuppressive responses by promoting differentiation into monocytic myeloid-derived suppressor cells. J. Immunol..

[61] Aurora AB, Olson EN (2014). Immune modulation of stem cells and regeneration. Cell Stem Cell..

[62] Nagy, T. *et al.* Hyperglycemia-induced aberrant cell proliferation; A metabolic challenge mediated by protein O-GlcNAc modification. *Cells.* **8,** (2019). 10.3390/cells8090999 PMC6769692 31466420

[63] Bolanle, I. O. & Palmer, T. M. Targeting protein O-GlcNAcylation, a link between type 2 diabetes mellitus and inflammatory disease. *Cells.* **11,** (2022). 10.3390/cells11040705 PMC8870601 35203353

[64] Bury JJ (2021). Type 2 diabetes mellitus-associated transcriptome alterations in cortical neurones and associated neurovascular unit cells in the ageing brain. Acta Neuropathol. Commun..

[65] Beckman JA (2019). Comparative transcriptomics of ex vivo, patient-derived endothelial cells reveals novel pathways associated with type 2 diabetes mellitus. JACC Basic Transl. Sci..

[66] Noreen, Z. *et al.* Transcriptional profiling and biological pathway(s) analysis of type 2 diabetes mellitus in a Pakistani population. *Int. J. Environ. Res. Public Health.* **17**, (2020). 10.3390/ijerph17165866 PMC7460550 32823525

[67] Mizoguchi E (2002). Role of tumor necrosis factor receptor 2 (TNFR2) in colonic epithelial hyperplasia and chronic intestinal inflammation in mice. Gastroenterology..

[68] Xiao T, Yan Z, Xiao S, Xia Y (2020). Proinflammatory cytokines regulate epidermal stem cells in wound epithelialization. Stem Cell Res. Ther..

[69] De Becker A, Riet IV (2016). Homing and migration of mesenchymal stromal cells: How to improve the efficacy of cell therapy?. World J. Stem Cells..

[70] Majidinia M, Aghazadeh J, Jahanban-Esfahlani R, Yousefi B (2018). The roles of Wnt/β-catenin pathway in tissue development and regenerative medicine. J. Cell Physiol..

[71] Li C, Li Z, Zhang Y, Fathy AH, Zhou M (2018). The role of the Wnt/β-catenin signaling pathway in the proliferation of gold nanoparticle-treated human periodontal ligament stem cells. Stem Cell Res. Ther..

[72] Onishi K, Zandstra PW (2015). LIF signaling in stem cells and development. Development..

[73] Santos GC (2020). Leukemia inhibitory factor (LIF) overexpression increases the angiogenic potential of bone marrow mesenchymal stem/stromal cells. Front. Cell Dev. Biol..

[74] Zhang Q (2012). Autophagy activation: A novel mechanism of atorvastatin to protect mesenchymal stem cells from hypoxia and serum deprivation via AMP-activated protein kinase/mammalian target of rapamycin pathway. Stem Cells Dev..

[75] Zhou P, Tan YZ, Wang HJ, Wang GD (2017). Hypoxic preconditioning-induced autophagy enhances survival of engrafted endothelial progenitor cells in ischaemic limb. J. Cell Mol. Med..

[76] Li ZH, Wang YL, Wang HJ, Wu JH, Tan YZ (2020). Rapamycin-preactivated autophagy enhances survival and differentiation of mesenchymal stem cells after transplantation into infarcted myocardium. Stem Cell Rev. Rep..

[77] Li H, Durbin R (2009). Fast and accurate short read alignment with Burrows-Wheeler transform. Bioinformatics.

[78] Kanehisa M, Goto S (2000). KEGG: Kyoto encyclopedia of genes and genomes. Nucleic Acids Res..

