# Supplementary material for: Identifying transcriptomic profiles of iron–quercetin complex treated peripheral blood mononuclear cells from healthy volunteers and diabetic patients
Source: Sci Rep. 2024 Apr 24;14:9441. doi: 10.1038/s41598-024-60197-1 (PMC11043337; doi:10.1038/s41598-024-60197-1)
Supplement: Supplementary file 1 — Supplementary Figures. [file 41598_2024_60197_MOESM1_ESM.docx]

**Supplementary information**

**The iron–quercetin complex induces alterations in the transcriptome profile of peripheral blood mononuclear cells, leading to enhanced biological function**

Phattarawadee Innuan^1,2^, Chonticha Sirikul^3^, Nampeung Anukul^3^, Gwenaël Rolin^4,5^, Nathupakorn Dechsupa^1,2^, Jiraporn Kantapan^1,2*^

**Supplementary Figure**


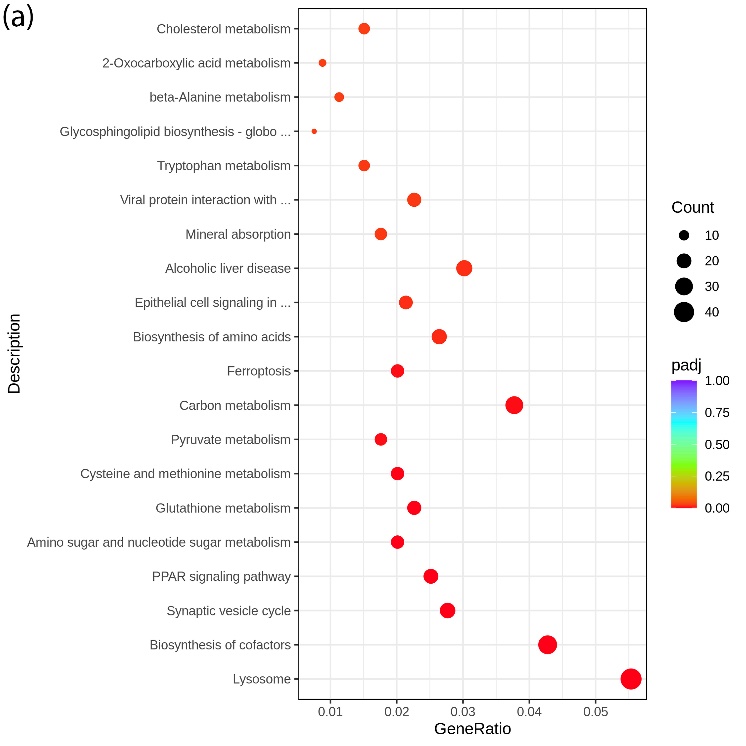

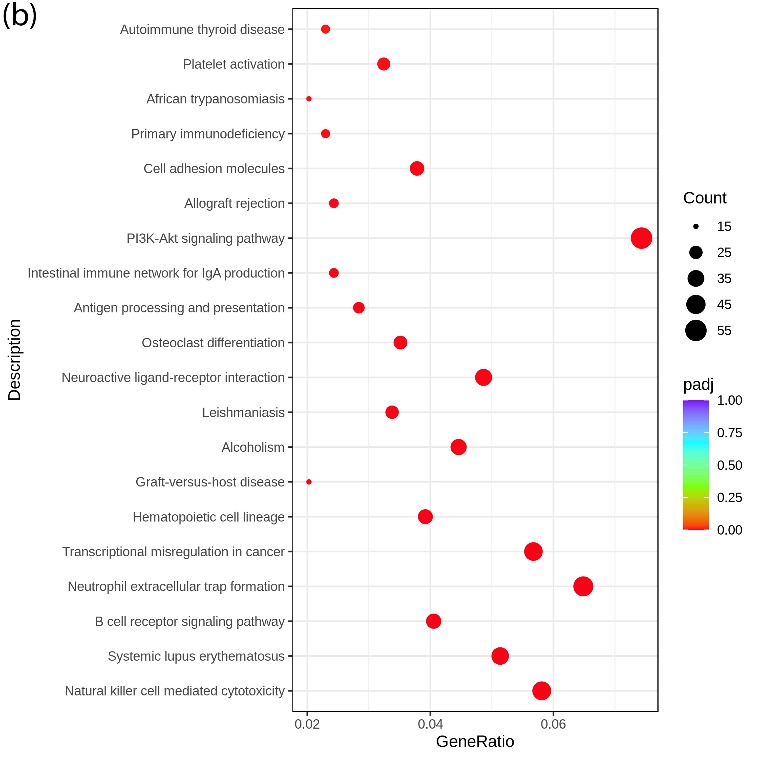


**Figure S1** The KEGG enrichment analysis of (a) upregulated and (b) downregulated DEGs between IronQ-treated PBMCs (NM-post) and untreated PBMCs (NM-pre) from healthy donors.


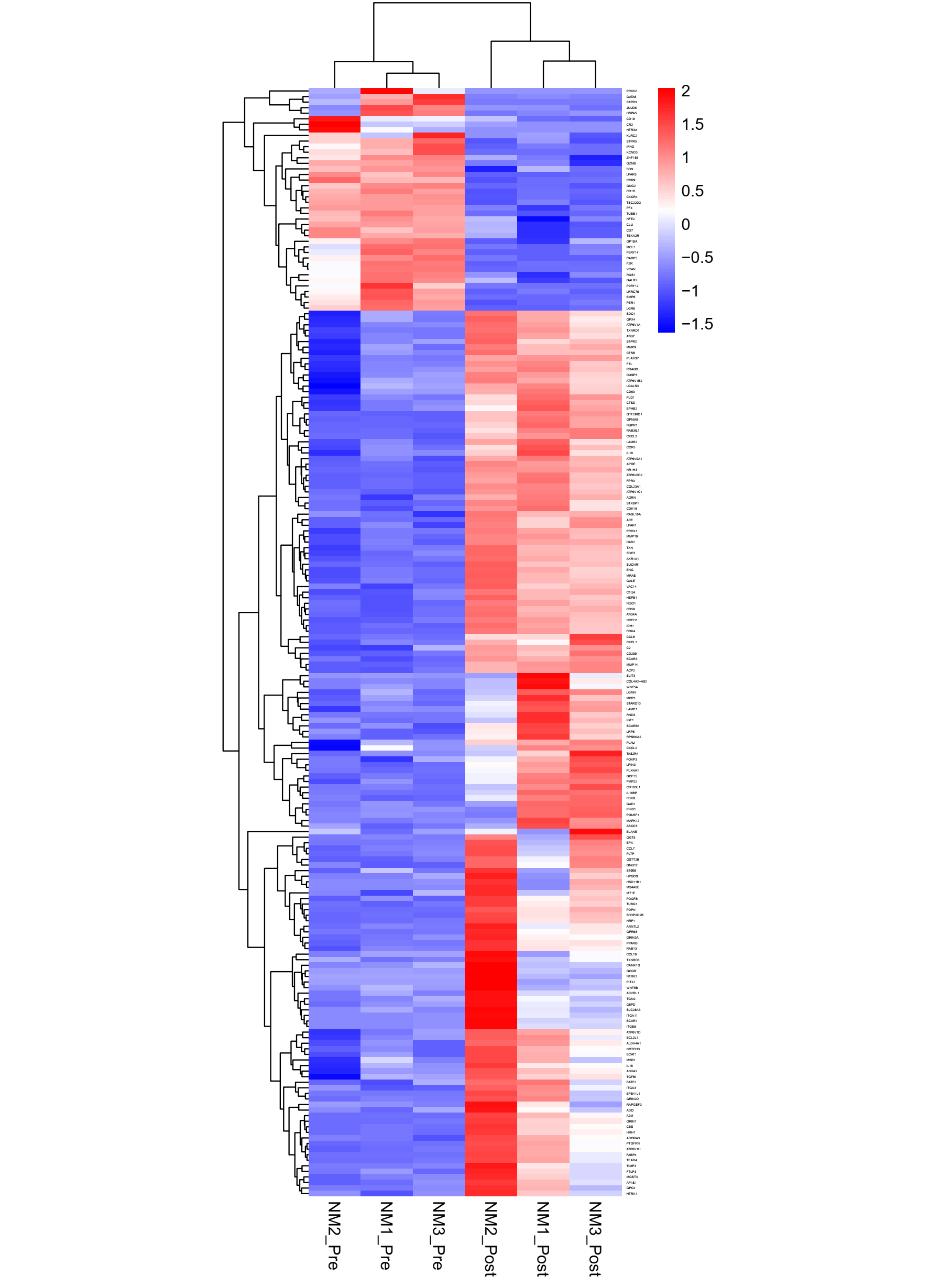


**Figure S2** Heatmap of the top 200 DEGs (ranked by p-value) in IronQ-treated Peripheral Blood Mononuclear Cells (PBMCs) from healthy donors.

**
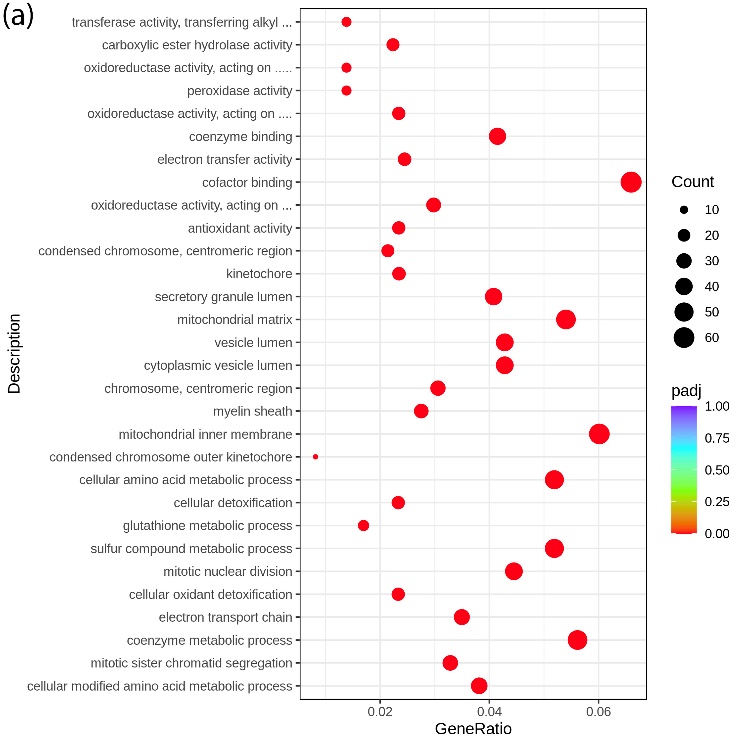

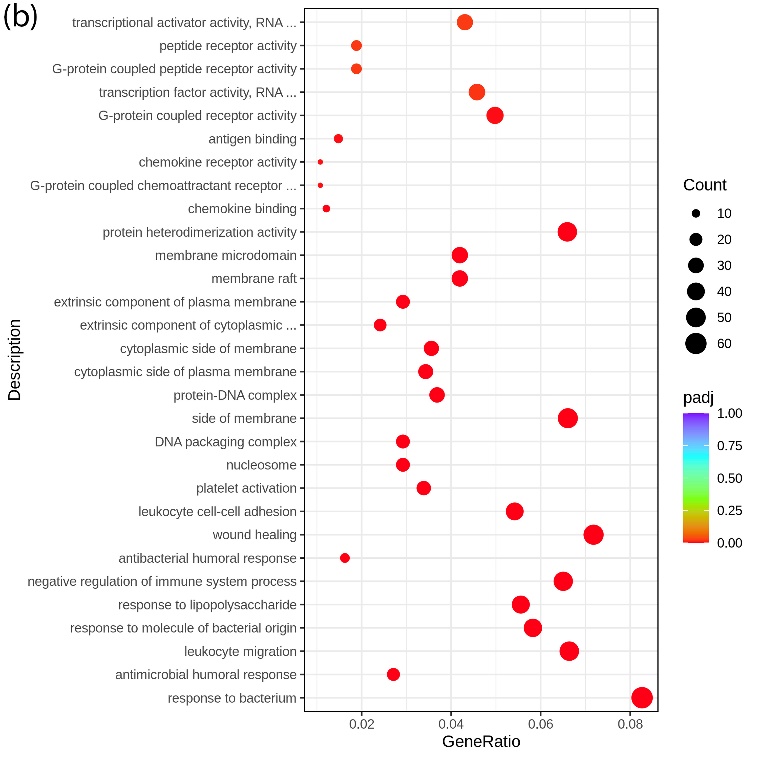
**

**Figure S3** The GO enrichment analysis of (a) upregulated and (b) downregulated DEGs between IronQ-treated PBMCs (DM-post) and untreated PBMCs (DM-pre) from diabetes donors.


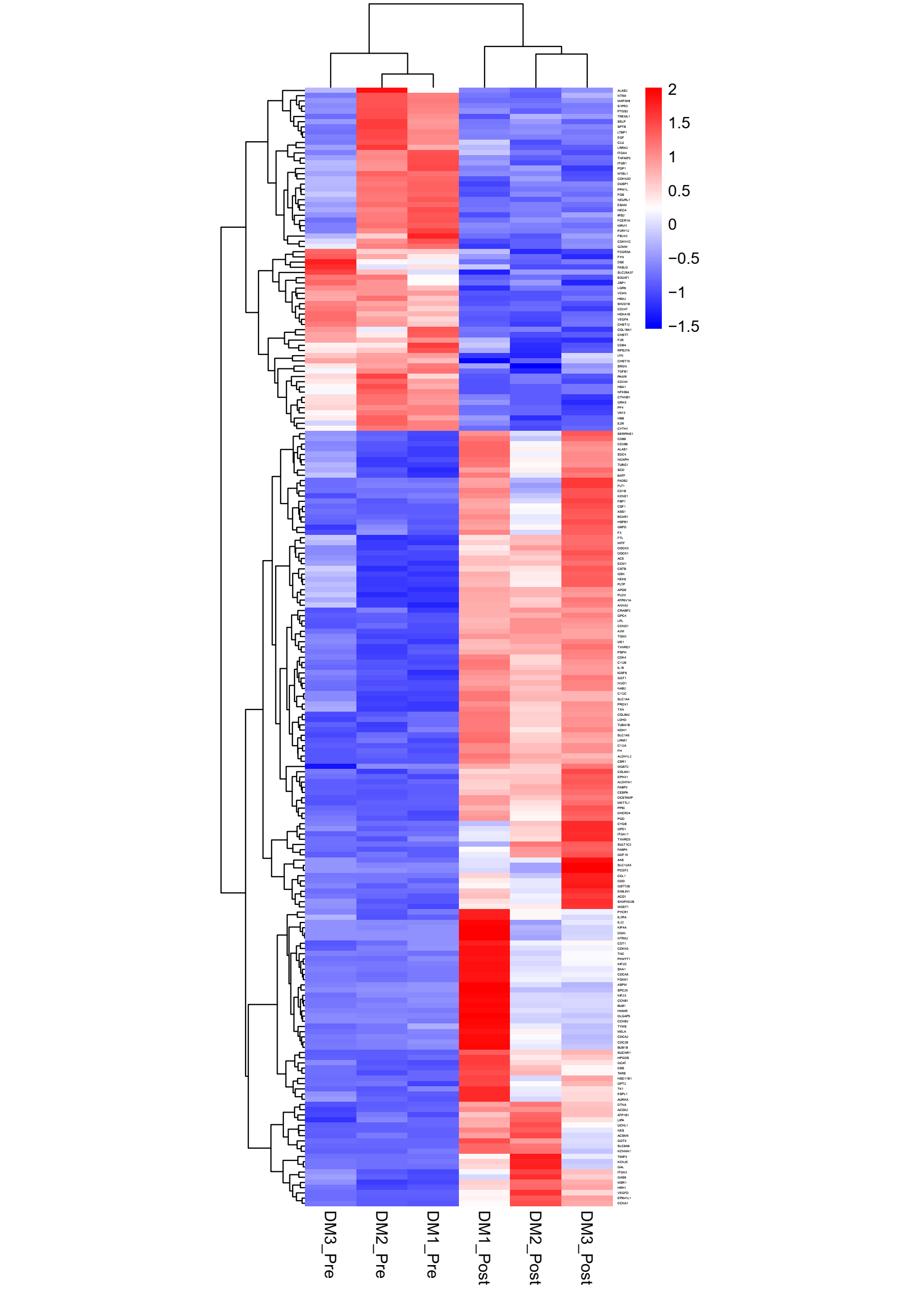


**Figure S4** Heatmap of the top 200 DEGs (ranked by p-value) in IronQ-treated Peripheral Blood Mononuclear Cells (PBMCs) from diabetes donors.

**
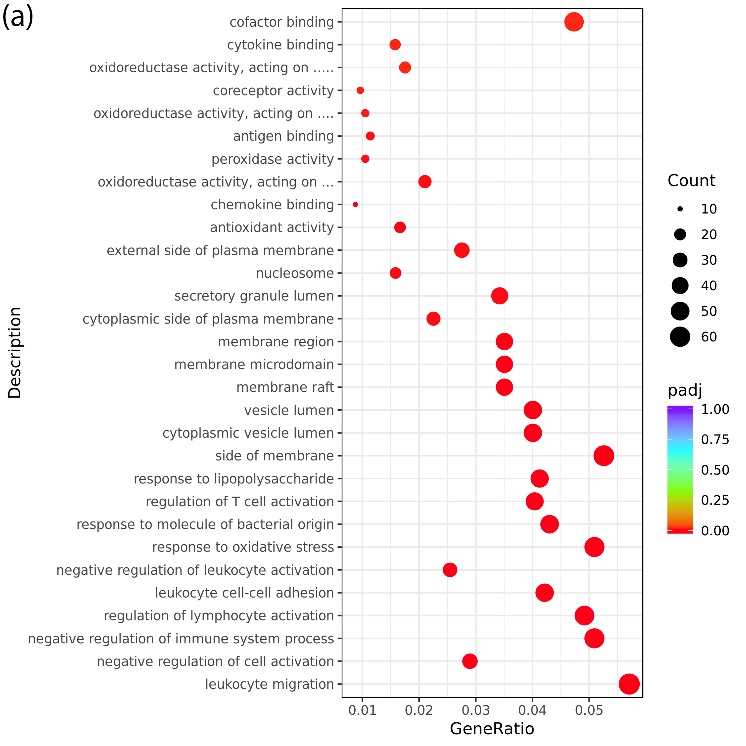
**

**
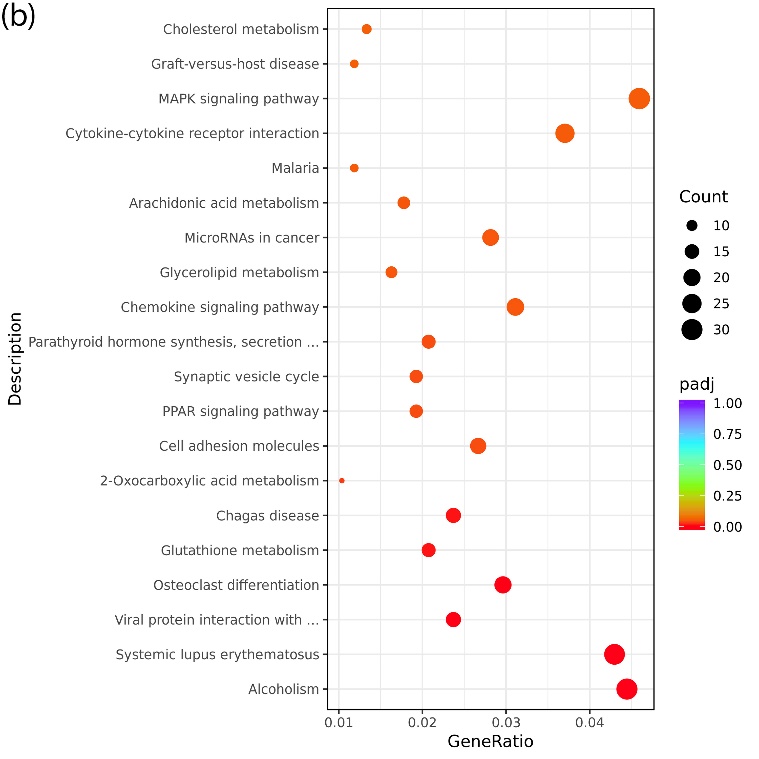
**

**
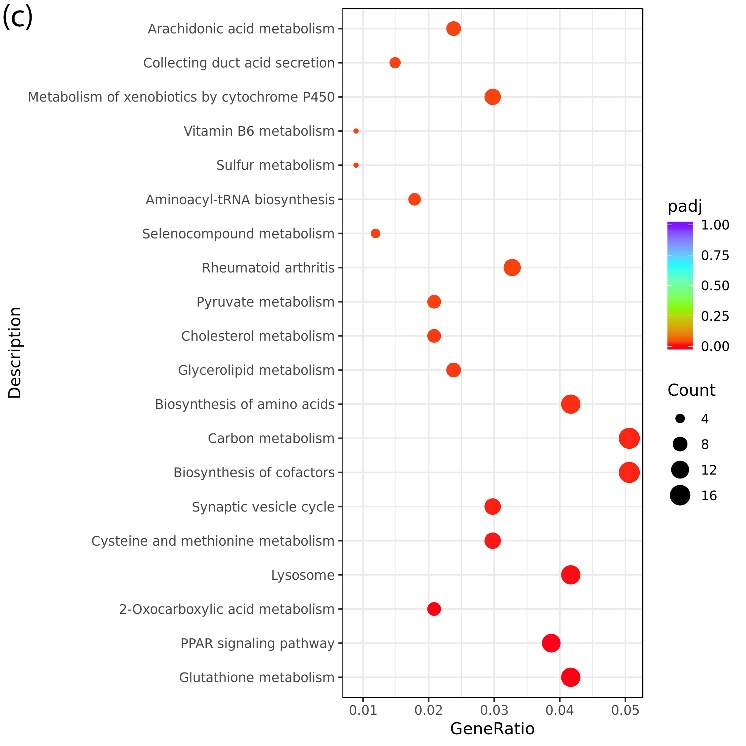

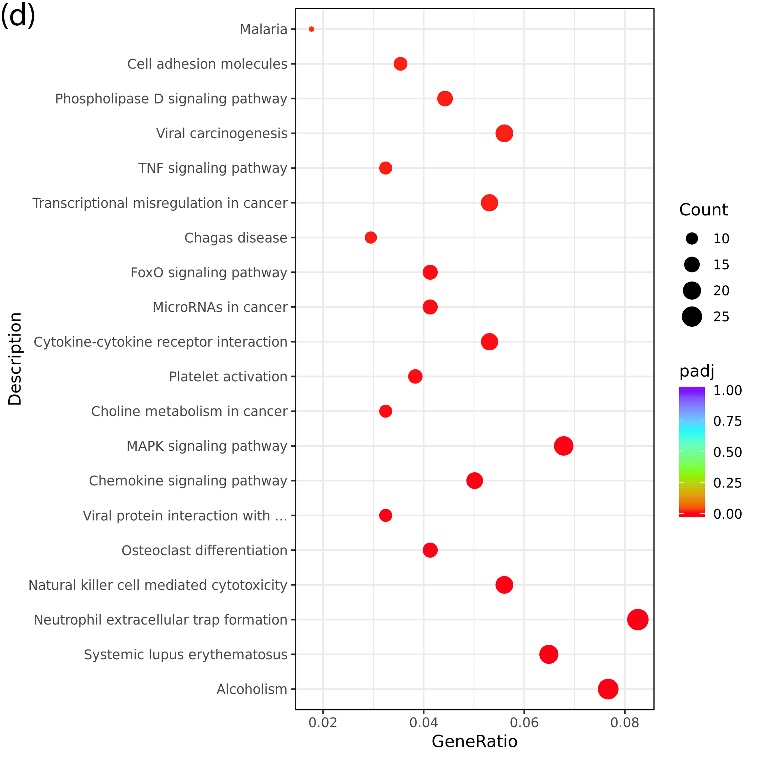
**

**Figure S5** (a) Gene Ontology (GO) enrichment analysis and (b) Kyoto Encyclopedia of Genes and Genomes (KEGG) enrichment analysis for all of the common DEGs in both healthy and diabetes donor PBMCs following treatment with IronQ. (c) KEGG enrichment analysis of only up-regulated and (d) only down-regulated common DEGs in both healthy and diabetes donor PBMCs following treatment with IronQ.

**
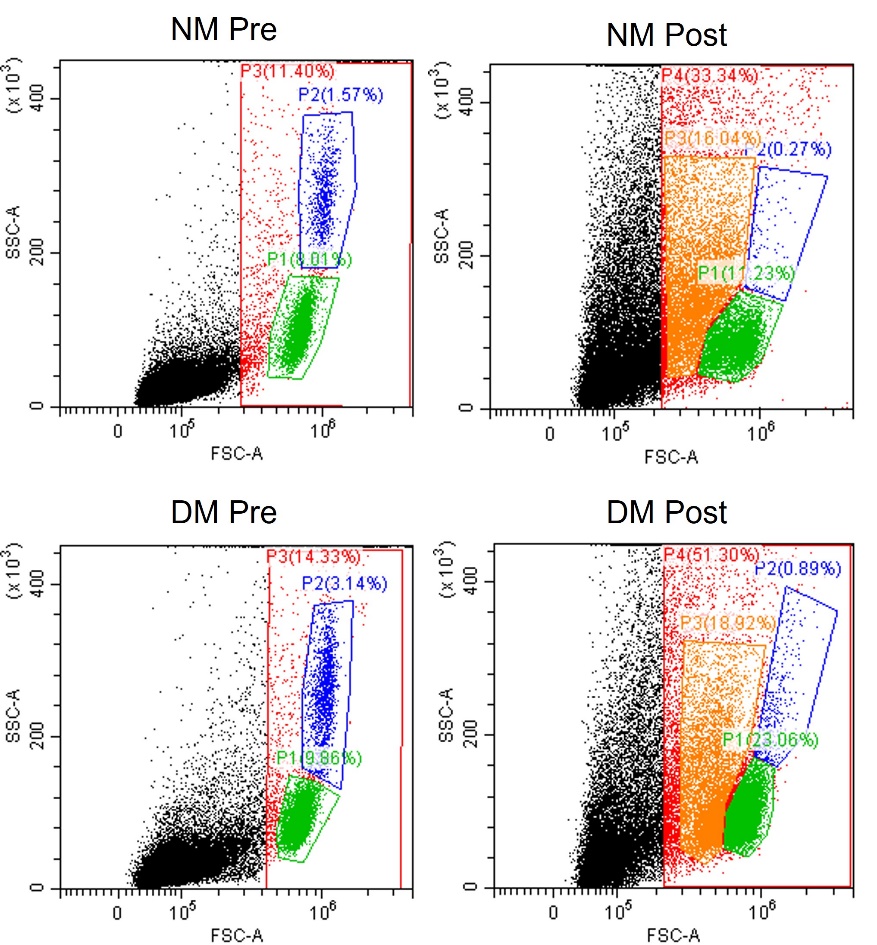
**

**Figure S6** Scatter plots representing flow cytometric analysis data, which reveal the comparability of subpopulations between PBMCs from healthy donors and diabetes donors, as well as the variations in subpopulations between pre-treatment and post-treatment conditions.
